# Thorough statistical analyses of breast cancer co-methylation patterns

**DOI:** 10.1186/s12863-022-01046-w

**Published:** 2022-04-15

**Authors:** Shuying Sun, Jael Dammann, Pierce Lai, Christine Tian

**Affiliations:** 1 Department of Mathematics, Texas State University, San Marcos, TX USA; 2 St. Stephen’s Episcopal School, Austin, TX USA; 3 Massachusetts Institute of Technology, Cambridge, MA USA; 4 Liberal Arts and Science Academy, Austin, TX USA

**Keywords:** Breast cancer, Co-methylation, Correlation analysis

## Abstract

**Background:**

Breast cancer is one of the most commonly diagnosed cancers. It is associated with DNA methylation, an epigenetic event with a methyl group added to a cytosine paired with a guanine, i.e., a CG site. The methylation levels of different genes in a genome are correlated in certain ways that affect gene functions. This correlation pattern is known as co-methylation. It is still not clear how different genes co-methylate in the whole genome of breast cancer samples. Previous studies are conducted using relatively small datasets (Illumina 27K data). In this study, we analyze much larger datasets (Illumina 450K data).

**Results:**

Our key findings are summarized below. First, normal samples have more highly correlated, or co-methylated, CG pairs than tumor samples. Both tumor and normal samples have more than 93% positive co-methylation, but normal samples have significantly more negatively correlated CG sites than tumor samples (6.6% vs. 2.8%). Second, both tumor and normal samples have about 94% of co-methylated CG pairs on different chromosomes, but normal samples have 470 million more CG pairs. Highly co-methylated pairs on the same chromosome tend to be close to each other. Third, a small proportion of CG sites’ co-methylation patterns change dramatically from normal to tumor. The percentage of differentially methylated (DM) sites among them is larger than the overall DM rate. Fourth, certain CG sites are highly correlated with many CG sites. The top 100 of such super-connector CG sites in tumor and normal samples have no overlaps. Fifth, both highly changing sites and super-connector sites’ locations are significantly different from the genome-wide CG sites’ locations. Sixth, chromosome X co-methylation patterns are very different from other chromosomes. Finally, the network analyses of genes associated with several sets of co-methylated CG sites identified above show that tumor and normal samples have different patterns.

**Conclusions:**

Our findings will provide researchers with a new understanding of co-methylation patterns in breast cancer. Our ability to thoroughly analyze co-methylation of large datasets will allow researchers to study relationships and associations between different genes in breast cancer.

**Supplementary Information:**

The online version contains supplementary material available at 10.1186/s12863-022-01046-w.

## Introduction

Breast cancer is both the second most commonly diagnosed form of cancer and the second leading cause of cancer-related death among US women [1]. Millions of women in the US have a history of breast cancer, and about one eighth of women are diagnosed with breast cancer at some point in their lives. Breast cancer has been associated with numerous inherited and environmental risk factors [2]. BRCA1 and BRCA2 are two of the most well-known genes whose mutations are linked with an increased risk of breast cancer. Breast cancer can also develop due to somatic genetic changes sparked by a wide variety of external factors such as smoking, radiation exposure, obesity, and alcohol consumption [2].

In addition to genetic changes, many publications have shown critical links between epigenetic changes and cancer development [3–5]. Epigenetics is defined as the study of heritable changes that affect gene expression without changing the actual DNA sequence [6]. A typical example of an epigenetic event is DNA methylation, which occurs when a methyl group (−CH_3_) is covalently added to a cytosine base in the dinucleotide 5′-CG-3′ [7]. A CG or CpG site refers to a cytosine base linked to a guanine base by a phosphate bond. A CpG island is usually defined as a chromosome region that is more than 200 base pairs long and has an average of > 50% CG sites in addition to an observed-to-expected CG site ratio of > 0.6 [8]. For the Illumina methylation array data, CpG islands are defined as regions greater than 500 base pairs (bps) with at least 55% GC content and the expected/observed CpG ratio greater than 0.65. CpG shores are about 2000 bps from islands; CpG shelves are about 4000 bps from islands [9].

Methylation patterns are known to influence gene functions in various ways; for example, methylation can lead to increased oncogenic cell growth, genomic instability, and cytosine to thymine transition mutations that prevent the expression of tumor suppressor genes [3, 7, 10]. In particular, changes in DNA methylation patterns have also been specifically linked with breast cancer development [5, 11, 12]. Co-methylation is defined as the similarity or the strong correlation of methylation signals between CG sites. In general, there are two main types of co-methylation: within-sample (WS) co-methylation and between-sample (BS) co-methylation [13, 14]. WS co-methylation refers to methylation patterns between consecutive or nearby sites in one chromosome region. BS co-methylation refers to the methylation similarity or correlation of CG sites (or genes) across various samples and in different genomic regions. Note, in this paper, we study BS co-methylation. To simplify our writing, we use co-methylation in the rest of this paper.

The previous study by Akulenko and Helms has demonstrated a high functional correlation between co-methylating genes in breast cancer samples, which suggests that co-methylation could help point to functional similarities between unknown genes in breast cancer [11]. Zhang and Huang have investigated co-methylation patterns in multiple cancers and their potential usefulness as biomarkers [15]. However, these previous studies are conducted on relatively small datasets of Illumina Human Methylation 27K array data. Analyses based on small datasets cannot show a complete picture of how specific DNA methylation changes affect the functions and interactions of genes. In addition, although some pan-cancer co-methylation analyses have been done by identifying common co-methylation clusters among multiple cancers [15, 16], no thorough research has been done for breast cancer co-methylation patterns yet.

In order to more thoroughly investigate co-methylation patterns in breast cancer, we will conduct a comprehensive co-methylation analysis of breast cancer methylation datasets consisting of 485,577 CG sites. We will focus on overall methylation patterns with relation to physical distance, sign (i.e., positive or negative correlations), and number of high correlations in normal and tumor datasets. We will also investigate specific CG sites whose co-methylation patterns change significantly between normal and tumor samples. Due to the large data size, the analysis is computationally challenging. However, our ability to analyze datasets of this size can provide researchers with a new and improved understanding of co-methylation patterns in breast cancer. Furthermore, new findings will allow researchers to establish relationships and associations between different genes in the future.

The novelty of our study lies in the following aspects. First, to the best of our knowledge, our paper is the first one that thoroughly analyzes and compares the negative co-methylation patterns in both tumor and matched normal samples. Second, our study thoroughly investigates the CG pairs whose co-methylation patterns change from normal to tumor cells by addressing specific questions listed in the Methods section. Third, we show that chromosome X (ChrX) co-methylation patterns are different from those of the autosomes in a number of important ways.

## Methods

In order to conduct the co-methylation study, we use publicly available data of 53 breast cancer patients that are alive from The Cancer Genome Atlas (TCGA). We download the Illumina human methylation 450K array data for 53 primary tumors and adjacent solid tissue normal samples. Each 450K dataset consists of the methylation signals (i.e., beta values) of 485,577 CG sites or probes. Next, we will summarize the three key analysis steps.

### Step 1: preprocessing data

We filter the available data based on the following criteria:

1. Remove 8233 probes/sites that have the same start and end positions.
2. Remove 397 chromosome Y CG sites because all samples are female.
3. Remove 85,468 CG sites with missing data (i.e., NA) in all 53 samples (i.e., in both tumor and normal samples) with 391,479 CG sites left.
4. Remove CG sites with 1 outlier that is outside of 3 times the interquartile range (IQR).
5. Remove the CG sites whose maximum and minimum methylation level differences are less than 0.05.
6. Only keep the CG sites with methylation signals in at least 80% of the samples (i.e., with ≥43 observations in both tumor and normal sample).
7. Remove 21 CG sites with duplicate chromosome positions.

After filtering based on the first two criteria, we have 476,947 CG sites left. After filtering based on all the above criteria, there are 272,990 (273K) CG sites left for downstream analysis. Note that the fourth criterion is used to remove the impact of outliers on co-methylation analysis. The fifth criterion is to ensure that there is a certain level of methylation variation among the selected CG sites while still keeping a reasonably large number of CG sites for further analysis.

### Step 2: calculating correlation coefficients for the Illumina 273K data

We study co-methylation patterns by analyzing the Pearson correlation coefficients between any two distinct CG sites based on their methylation levels. A correlation coefficient of 1 or close to 1 would mean that the two CG sites are highly positively related; CG sites with a correlation coefficient of − 1 or close to − 1 are highly negatively related. This correlation calculation generates a large 272,990 by 272,990 matrix of correlation coefficients. However, R’s relatively limited storage capacity is exceeded by the size of a 272,990 by 272,990 correlation matrix file. Since the correlation matrix has to be stored in R in order to perform further analyses, we overcome the challenge of R’s limited storage using a divide and conquer strategy. We generate the correlation matrix in separate blocks in order to prevent the storage issue when the files are being generated. That is, we generate 273 files; each of them contains 1000 rows and 272,990 columns of correlation coefficients. We also truncate all the correlation coefficients to 4 decimal places in order to further save space. By dividing up the correlation matrix and truncating the correlation coefficients, we overcome the issue of R’s limited storage.

### Step 3: identifying co-methylation patterns using correlation analysis

We further analyze co-methylation patterns using the correlation coefficient between each pair of CG sites. We investigate the co-methylation patterns in both tumor and normal datasets and then compare them. When investigating the co-methylation patterns, we focus on addressing the following seven questions: (1) For each CG site, how many CG sites are highly correlated with it, and what is the distribution of these counts? (2) For those highly correlated CG sites located on the same chromosome, how far away are they from each other? That is, what is the distance distribution for co-methylated CG sites on the same chromosome? (3) What are the signs (positive or negative) of these high correlations? Are there any differences between normal and tumor samples? (4) Are there pairs of CG sites that have a large change in correlation between normal and tumor? If so, do they possess special qualities not seen in the overall datasets? (5) What patterns are present in these highly changing CG sites that are also differentially methylated? (6) Are there specific genes that are more closely related to these highly changing CG sites? If so, what are they, and what interactions do they have? (7) What genes are associated with the super-connector CG sites (sites highly correlated with a large number of other sites), and what interactions do these genes have?

## Results

### Overall co-methylation patterns

We determine co-methylated CG pairs to be those with a correlation coefficient greater than or equal to 0.8. This cutoff is used in a previous publication [11]. There are ${C}_2^{272990}$ = 37,261,633,555 possible pairs in both tumor and normal data, of which 298,194,565 in tumor and 794,262,245 in normal are co-methylated (i.e., highly correlated based on the 0.8 cutoff value). These give proportions of 0.80 and 2.13% in tumor and normal respectively, such that normal samples have roughly 2.7 times the high correlations in tumor samples, see Table 1.

**Table 1:** CG pairs with a high correlation level

	Total CG Sites	Total CG Pairs	Pairs with |Correlation| ≥ 0.8	Proportion with |Correlation| ≥ 0.8
Normal	272,990	37,261,633,555	794,262,245	2.13%
Tumor	272,990	37,261,633,555	298,194,565	0.80%

**Fig. 1:**
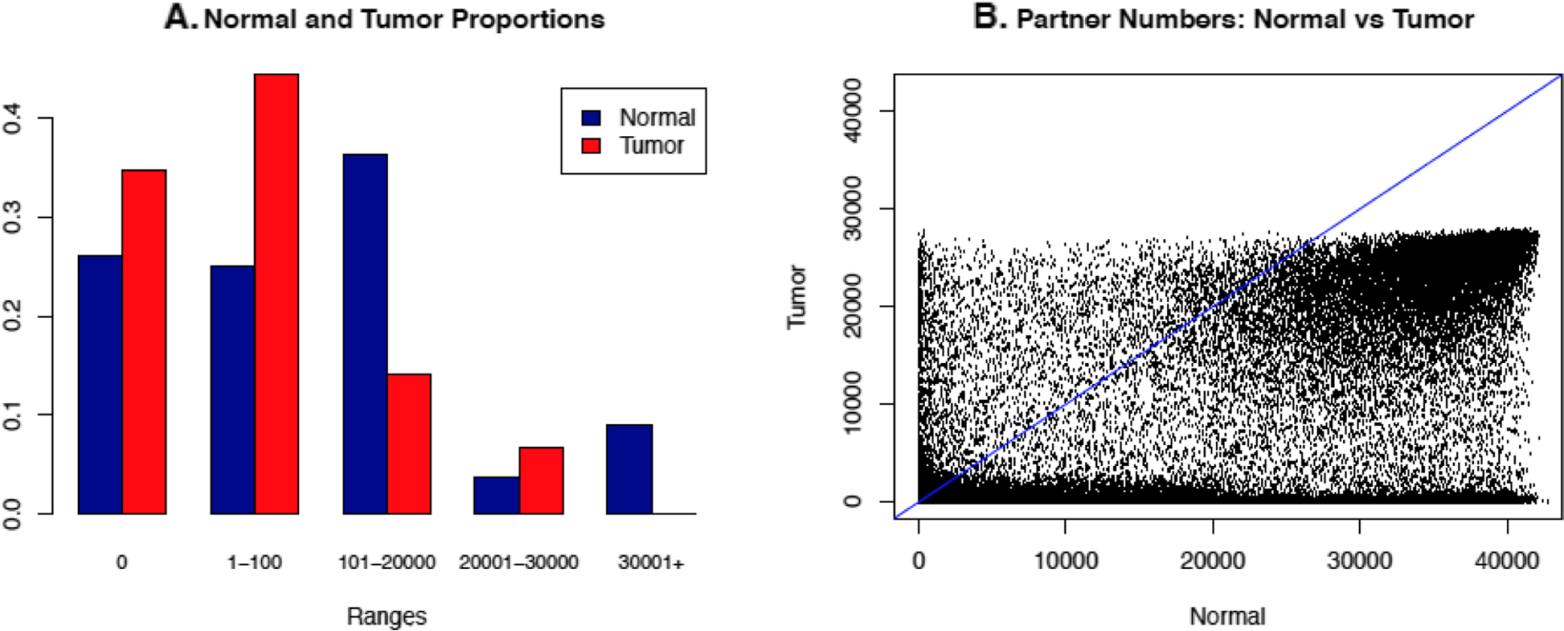
Trends regarding highly correlated CG pairs in normal and tumor data. **A** shows a general trend of the number of CG sites that each CG site is correlated with. The first two bars show that less than 30% of CG sites in the normal data are not highly correlated with any other CG sites, but more than 30% of CG sites in the tumor data are not highly correlated with other CG sites. **B** is a scatterplot comparing tumor and normal correlations. For **B**, the x-axis represents the number of CG sites a specific CG site is highly correlated with in the normal data, and the y-axis represents the number of CG sites a specific CG site is highly correlated with in the tumor data

For all ${C}_2^{272990}$ possible pairs, Table 2 shows the summary of the number of CG sites that each CG site is highly correlated with. The distributions are extremely skewed to the right, with most sites highly correlated with a few CG sites and a small number of sites correlated with a lot of CG sites. Note that the numbers for normal samples are generally higher than the numbers for tumor samples. Furthermore, Fig. 1A shows that the tumor dataset has more CG sites with a number of correlations under 100 compared to the normal dataset. The normal dataset has more in the 101 to 20,000 range. After 30,000, the tumor dataset drops to 0 (as shown in Table 2, the maximum in the tumor dataset is 27,996 correlations), so the normal dataset has more correlations in that range. A bar graph with more groups can be seen in the Supplemental Fig. 1 in the Additional file 1.

**Table 2:** Summary for the number of CG sites each CG site is highly correlated with

	Min	Q1	Median	Mean	Q3	Max
Normal	0	0	84	5819	4375	42,787
Tumor	0	0	3	2185	50	27,996

Q1 and Q3 mean 25^th^ and 75^th^ percentiles respectively

Figure 1B is a scatterplot that compares the number of high correlations each CG site has in the tumor data and the normal data. The blue line is for scale only; it has a slope of 1. As we see, there is a dark line along the x-axis, which corresponds to a large number of CG sites having few highly correlated partners in tumor but many such partners in normal. This pattern also exists along the y-axis, though to a much lesser extent. In addition, there is a large clump in the top right-hand corner, indicating that many CG sites have high correlations with a lot of sites in both normal and tumor data. We also note that the plot has a surprisingly well-defined border; the number of points appears to drop significantly at around x = 42,000 and y = 27,000, which are the rough maximum number of CG sites a specific CG site can highly correlate with in normal and tumor respectively.

### Co-methylation signs and chromosome patterns

We further examine the overall correlation patterns based on if the two CG sites in each pair are on the same or different chromosomes and if the correlation is positive or negative. The overall statistics are in Table 3. We see that the vast majority of CG pairs are on different chromosomes (about 94% for both tumor and normal) and are positively correlated (93.4% for normal and 97.19% for tumor). Note that there are two striking patterns. First, although the percentages in normal and tumor are similar, the number of pairs can be dramatically different. For example, for the pairs on the same chromosome, there are 45.2 million in the normal dataset and 17.5 million in the tumor dataset. That is, the number of normal pairs is about 2.5 times the number of tumor pairs. Second, for the pairs with negative correlations, normal samples have more negative pairs than tumor samples (6.6% for normal vs. 2.81% for tumor), and the two-proportion test *p*-value is < 2.2 × 10^− 16^, which shows that tumor and normal datasets are significantly different. For the pairs with positive correlations, tumor samples seem to have a relatively larger percentage (93.4% for normal and 97.19% for tumor), but the total number of positive pairs is much less than the number in the normal data (741.9 million for normal and 289.8 million for tumor).

**Table 3:** Co-methylated CG pairs on the same/different chromosomes and with positive/negative correlations

	Total Pairs	Pairs on Same Chr.	Pairs on Diff. Chr.	Negative Pairs	Positive Pairs
Normal	794,262,245	45,249,327 (5.70%)	749,012,918 (94.30%)	52,400,697 (6.60%)	741,861,548 (93.40%)
Tumor	298,194,565	17,490,793 (5.87%)	280,703,772 (94.13%)	8,391,729 (2.81%)	289,802,836 (97.19%)

**Fig. 2:**
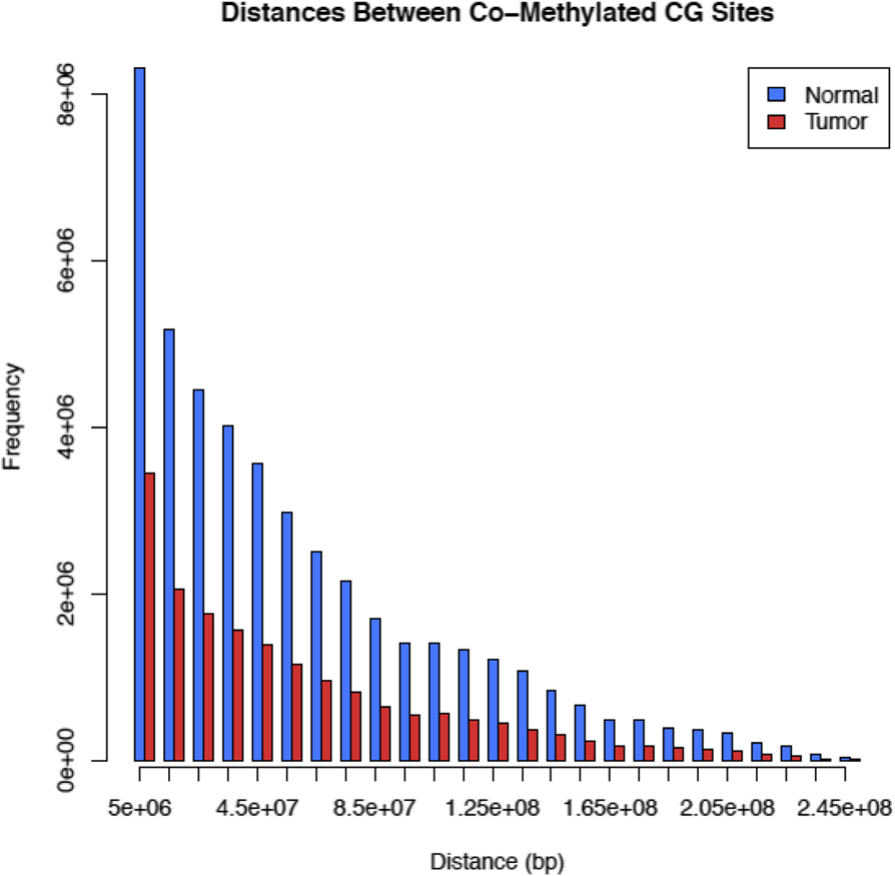
Distances between co-methylated CG pairs on the same chromosome

For the highly correlated pairs that are on the same chromosome, we examine the distribution of the distances between the CG sites in each pair in terms of base pairs (see Fig. 2). Due to the fact that there are almost three times as many total normal pairs as there are tumor pairs, every interval of ten million base pairs has more normal pairs than tumor pairs. The tumor and normal counts for these intervals are significantly different with *p*-value = 8.895 × 10^− 5^. However, the distribution of distances between co-methylating CG sites is relatively similar. We find an inverse association between co-methylation and genomic distance; that is, co-methylation is more likely to occur between CG pairs that are located close to each other.

A further analysis shows that the median distance among all pairs on the same chromosome is 39,617,206 for tumor and 41,737,711 for normal, which is about a 2 million base difference (see Table 4). We perform t-tests on the distances between highly correlated CG sites for each chromosome to compare tumor samples with normal samples. Test results show that there is a significant difference (with extremely small *p*-values close to 0) for all chromosomes, though this finding is likely due to very large distances and a large number of sites (see Supplemental Table 1 in the Additional file 1). We therefore also examine distances between pairs for each single chromosome using side-by-side boxplots for tumor and normal data. The vast majority of chromosomes exhibit distance patterns similar to that of the overall data. That is, although the mean or median differences are relatively large, the boxplots do not clearly illustrate this difference because there is a large range as seen in the whole genome data. However, ChrX exhibits a significantly different pattern, where the median distance between tumor CG pairs is much smaller than the median distance between normal CG pairs, meaning that the tumor co-methylated CG pairs are concentrated more closely together than the normal co-methylated CG pairs. The overall shape of the histograms of distances between correlated CG site pairs on the same chromosome appears to be mostly the same between normal and tumor data, with only the ChrX distances being notably different. Later, we further study the co-methylation patterns on ChrX separately, and the results are summarized in the subsection titled “Chromosome X.”

**Table 4:** Summary of the distances between co-methylated CG pairs on the same chromosome

	Min	Q1	Median	Mean	Q3	Max
Tumor	2	14,111,897	39,617,206	54,455,914	80,313,554	248,046,750
Normal	2	15,498,757	41,737,711	57,065,415	84,514,828	248,094,980

**Fig. 3:**
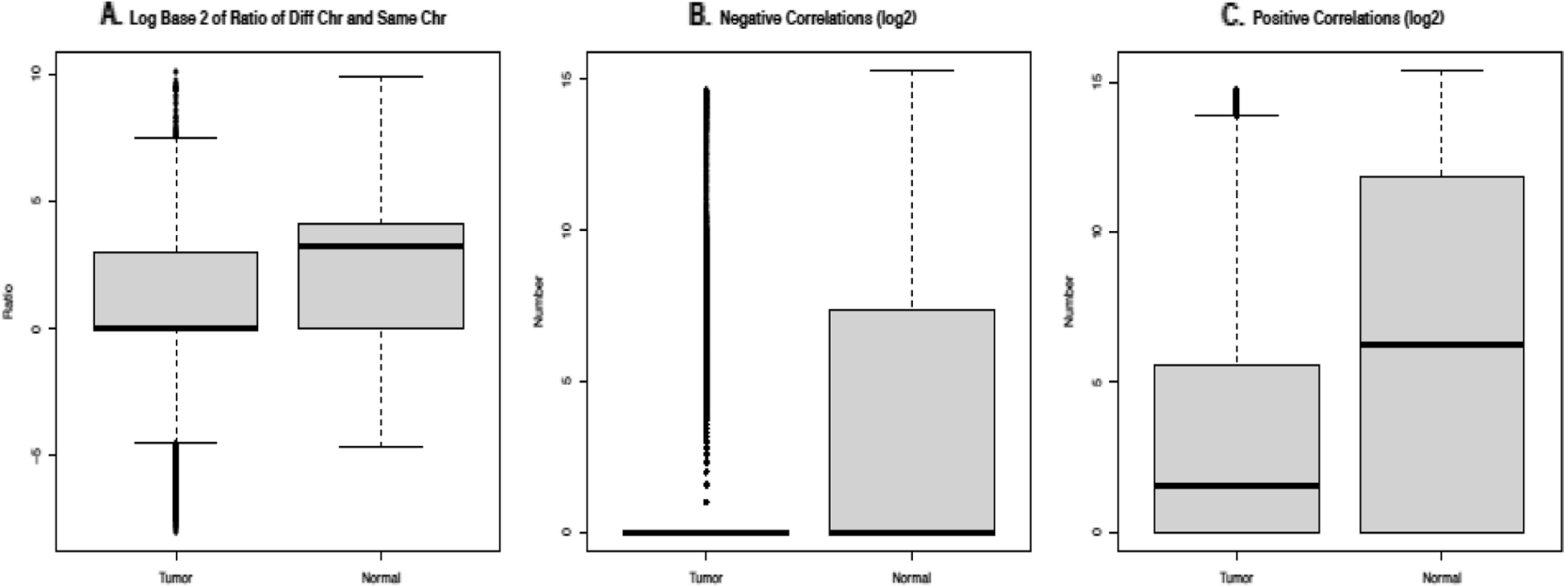
Boxplots of co-methylation signs and chromosome patterns. **A** Boxplot of ${\mathit{\log}}_2\left(\frac{diff+1}{same+1}\right)$ for each CG site in the tumor and normal datasets. **B** Boxplot of the number of negatively correlated CG sites. **C** Boxplot of the number of positively correlated CG sites. Note, log_2_(negative correlations+1) and log_2_(positive correlations+1) are used for **B** and **C**

We also plot the number of CG pairs on the same or different chromosomes, see the top two plots of Supplemental Fig. 2 in the Additional file 1. In these figures, the dots appear to form lines, suggesting that a lot of the CG sites have the same ratio of same to different chromosome correlations. We also see that the slope of the lines is much less than one, which is expected as the ratio of same to different chromosome correlations is very low. In order to see the pattern clearly, we plot log_2_((diff + 1)/(same + 1)) as shown in Fig. 3A, where “diff” means the number of highly correlated CG partners on different chromosomes and “same” means the number of CG partners on the same chromosome. The additional 1 added to each of the numbers (i.e., “diff+ 1” and “same+ 1”) is to account for CG sites that are highly correlated with no other CG sites. This is to avoid calculating log_2_(0). For the tumor dataset, we can see that the median value is at 0, while in the normal dataset, the median is greater than 0. This indicates that in the tumor dataset, the numbers of same and different chromosome pairs are more likely to be equal to each other, while in the normal dataset, there tends to be more CG pairs that are on different chromosomes. The tumor dataset also has a noticeably larger number of outliers than the normal dataset, indicating more heterogeneity within the tumor dataset.

As for the positive/negative correlation patterns, there appear to be a lot of CG sites with a high number of negative correlations or positive correlations in either normal or tumor, but not both, as shown in Supplemental Fig. 2 in the Additional file 1 bottom plots’ horizontal and vertical axes. In the normal graph, there is also a fairly large clump of points with a significant number of both negative and positive correlations. In addition, we also plot log_2_(number of positive correlations + 1) and log_2_(number of negative correlations + 1) for both the tumor and normal data in Fig. 3B and C. The additional 1 added to each of the numbers is to account for CG sites that are highly correlated with no other CG sites. This is to avoid calculating log_2_(0). For the negative correlations, the tumor dataset has a much larger number of outliers, which again indicates more heterogeneity within the tumor dataset. When looking at the positive correlations, there are also more outliers in the tumor dataset than in the normal dataset. In the positive correlation boxplots, the median of the normal dataset is also noticeably higher than the median of the tumor dataset. This difference suggests that the CG sites in the normal dataset tend to form more positive correlations than the sites in the tumor dataset. Finally, paired t-tests are conducted for data shown in Fig. 3A, B, and C to compare tumor and normal samples. Each paired t-test yields an extremely small *p*-value of almost 0, which shows the differences are statistically significant.

**Fig. 4:**
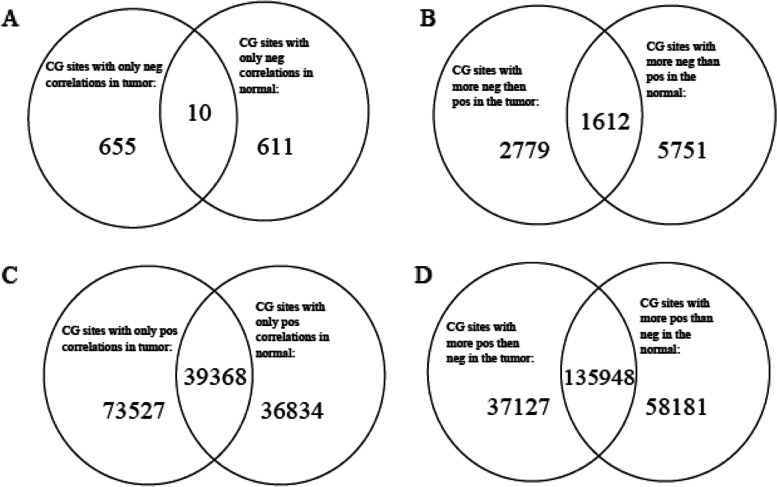
Detailed analysis of positive and negative co-methylation patterns. **A** Venn diagram of the number of CG sites that only have negative correlations. **B** Venn diagram of the number of CG sites that have more negative correlations than positive correlations. **C** Venn diagram of the number of CG sites that only have positive correlations. **D** Venn diagram of the number of CG sites that have more positive correlations than negative correlations

We find that the percentage of negative correlations is significantly different between normal (6.6%) and tumor (2.8%) as shown in Table 3. We then identify the following CG sites: A. CG sites that only have negative correlations with other CG sites; B. CG sites that have more negative than positive correlations with other sites. We identify these two lists of CG sites in normal and tumor data separately and compare them; see the two top Venn diagrams in Fig. 4, i.e., A and B. Figure 4A shows that there are 621 CG sites in the normal dataset that only have negative correlations, and there are 665 CG sites in the tumor dataset that only have negative correlations. There are 10 CG sites out of > 600 CG sites that are overlapped between the tumor and normal. This finding tells us that different sets of CG sites in the tumor and normal datasets play certain roles by negatively correlating with other CG sites or genes. Figure 4B shows that there are 4391 CG sites in the normal and 7363 CG sites in the tumor that have more negative correlations than positive correlations. There are 1612 CG sites that overlap. Unlike the data shown in Fig. 4A, there seems to be a larger difference between the normal and tumor dataset when looking at the number of CG sites that have more negative than positive correlations.

We also compare positive correlations between the normal (93.40%) and tumor (97.19%) datasets. Figure 4C shows that there are 112,895 CG sites in the tumor dataset that only have positive correlations and that there are 76,202 CG sites in the normal dataset that only have positive correlations. There are 39,368 CG sites that are overlapped between the tumor and normal data. These overlapping CG sites make up 51.66% of the CG sites that only have positive correlations in the normal dataset and 34.87% of the CG sites that only have positive correlations in the tumor dataset. This is unlike the data shown in Fig. 4A, which shows a much smaller percentage of overlapping CG sites that only have negative correlations. Figure 4D shows that there are 173,075 CG sites in the tumor dataset that have more positive than negative correlations, and there are 194,129 CG sites in the normal dataset that have more positive than negative correlations. There are 135,948 overlapping CG sites. Finally, for each Venn diagram in Fig. 4, we compare the proportion of CG sites in tumor-only with the ones in normal-only data. The proportion-test *p*-values for Fig. 4B, C, and D are all extremely small (*p*-value < 2.2 × 10^− 16^). That is, there are significantly different proportions of CG sites that function in certain ways in tumor-only or normal-only cells. In addition to the proportion difference, all four Venn diagrams show different sets of CG sites that function differently in either tumor or normal samples.

### Highly changing CG pairs

To further investigate the relationship between the tumor and normal samples, we examine how the correlation coefficient of each CG pair changes by comparing the normal with the tumor samples. Following the example of a previous publication [17], we split the correlation coefficient values, all between − 1 and 1, into 8 intervals: [− 1, − 0.75), [− 0.75, − 0.50), [− 0.50, − 0.25), [− 0.25,0), [0, 0.25), [0.25, 0.5), [0.5, 0.75), [0.75, 1). The number of CG pairs that fall within certain intervals in the tumor and normal datasets are recorded in Table 5.

**Table 5:** The number of CG pairs whose correlation coefficients fall within certain intervals

	Tumor [− 1, − 0.75)	Tumor [− 0.75, − 0.50)	Tumor [− 0.50, − 0.25)	Tumor [− 0.25, 0)	Tumor [0, 0.25)	Tumor [0.25, 0.50)	Tumor [0.50, 0.75)	Tumor [0.75, 1)
Normal [−1, −0.75)	9,047,541 (8.530940%)	13,685,670 (12.904238%)	**21,792,226** **(20.547922%)**	**34,659,212** **(32.680221%)**	**22,030,844** **(20.772915%)**	4,588,346 (4.326358%)	249,385 (0.235145%)	2398 (0.002261%)
Normal [−0.75, −0.50)	4,532,553 (0.483943%)	43,143,743 (4.606482%)	139,239,351 (14.866663%)	**343,557,340** **(36.681809%)**	**325,165,891** **(34.718144%)**	76,117,608 (8.127120%)	4,773,392 (0.509658%)	57,889 (0.006181%)
Normal [−0.50, −0.25)	950,340 (0.026118%)	30,082,357 (0.826755%)	321,883,396 (8.846337%)	**1,289,397,068** **(35.436564%)**	**1,551,479,465** **(42.639387%)**	419,567,332 (11.530990%)	24,952,455 (0.685770%)	293,958 (0.008079%)
Normal [−0.25, 0)	392,148 (0.004785%)	17,342,879 (0.211636%)	385,387,947 (4.702910%)	**2,635,151,893** **(32.156900%)**	**3,855,226,940** **(47.045542%)**	1,221,099,246 (14.901140%)	79,112,772 (0.965417%)	956,285 (0.011670%)
Normal [0, 0.25)	198,005 (0.001766%)	11,092,196 (0.098906%)	312,990,583 (2.790851%)	**2,905,279,677** **(25.905581%)**	**5,662,488,508** **(50.490855%)**	2,156,699,767 (19.230700%)	163,940,452 (1.461812%)	2,190,125 (0.019529%)
Normal [0.25, 0.50)	70,324 (0.000822%)	4,876,128 (0.056974%)	150,373,427 (1.756992%)	1,709,044,540 (19.968805%)	**4,212,522,101** **(49.219918%)**	**2,235,224,229** **(26.116789%)**	240,956,009 (2.815376%)	5,505,226 (0.064324%)
Normal [0.50, 0.75)	16,019 (0.000457%)	1,236,027 (0.035233%)	30,003,186 (0.855234%)	451,563,452 (12.871708%)	**1,493,350,206** **(42.567591%)**	**1,177,953,830** **(33.577292%)**	304,867,905 (8.690187%)	49,195,266 (1.402299%)
Normal [0.75, 1)	1171 (0.000106%)	101,571 (0.009200%)	2,299,244 (0.208250%)	36,610,029 (3.315896%)	**223,487,199** **(20.242003%)**	**303,425,075** **(27.482251%)**	212,705,866 (19.265501%)	**325,446,342** **(29.476793%)**

The sum of all the cells in the table is 272,990 × 272,899 / 2 = 37,249,349,005. The percentages refer to the number of pairs in the cell out of the total number of pairs in the row. For example, the 8.53% in the top-left-most cell means that 9,047,541 out of 106,055,622 CG pairs (i.e., 8.53%) have a correlation within [− 1, − 0.75) in both tumor and normal datasets. Most of the CG pair correlation values do not change much between the normal and the tumor dataset, especially when the correlation value in the normal dataset is in the range [− 0.5, 0.5]. The largest CG percentages for each row, those being 20-50%, are either found to have normal and tumor correlation values that are within the same range or values that are separated by 1-2 intervals. The percentages that are > 20% are in bold.

There are 3569 highly changing CG pairs that cross 7 intervals. Among these CG pairs, 2398 of them change from very high negative correlations (i.e., [− 1.0, − 0.75)) in the normal data to very high positive correlations (i.e., [0.75, 1.0]) in the tumor data, and 1171 CG pairs change from very high positive correlations ([0.75, 1.0]) in the normal data to very high negative correlations ([− 1.0, − 0.75)) in the tumor data. Further examination of these 3569 CG pairs reveals that there are 1443 unique CG sites that are involved in the negative to positive changes and 822 unique CG sites that are involved in the positive to negative changes. The union of these two sets has only 1880 unique CG sites, so there is a considerable overlap; that is, 385 CG sites are involved in the changes in both directions. We then separate the 1880 unique CG sites involved in the highly changing CG pairs into three different groups as shown in Table 6. 385 CG sites that are involved in both the positive to negative and negative to positive changes form the “both.direction” group. 437 CG sites that are only involved in the positive to negative changes form the “uniq.pos2neg” group. Lastly, 1085 CG sites that are only involved in the negative to positive changes form the “uniq.neg2pos” group. We will study the DM patterns of these CG sites in the next section.

**Table 6:** DM Statuses of CG sites involved in co-methylation changes between normal and tumor data

	#CG Sites	#DM	%DM
both.direction	385	50	12.99%
uniq.pos2neg	437	47	10.76%
uniq.neg2pos	1058	101	9.55%
All	272,990	23,361	8.56%

### Differential methylation

Next, we investigate whether there is any relationship between co-methylation and differential methylation. In particular, we study how many of the CG sites sorted into the three groups mentioned previously are DM, meaning there is a significant difference between their normal and tumor methylation levels. We perform paired t-tests on the 53 methylation levels between normal and tumor for all 272,990 CG sites. To be considered a DM site, the *p*-value of the t-test must be < 0.05, and the absolute mean difference value must be > 0.2. Information on the number of CG sites involved in the co-methylation changes and the number of DM CG sites can be found in the last two columns of Table 6. Among all the highly-changing CG sites, 9.55-12.99% are DM sites. These percentages are larger than the overall DM rate, which is 8.56%. The CG sites highly changed in the both.direction group have a very large difference compared with the average DM rate (12.99% vs. 8.56%). We also look at the number of DM CG sites by chromosome (See Supplemental Fig. 3 and Supplemental Table 2 in the Additional file 1). For Chr1 to Chr22, the percentages of DM CG sites fall within the 5.91-11.75% range. ChrX, however, only has 2.22% DM sites, marking another way in which ChrX differs from the other chromosomes.

**Fig. 5:**
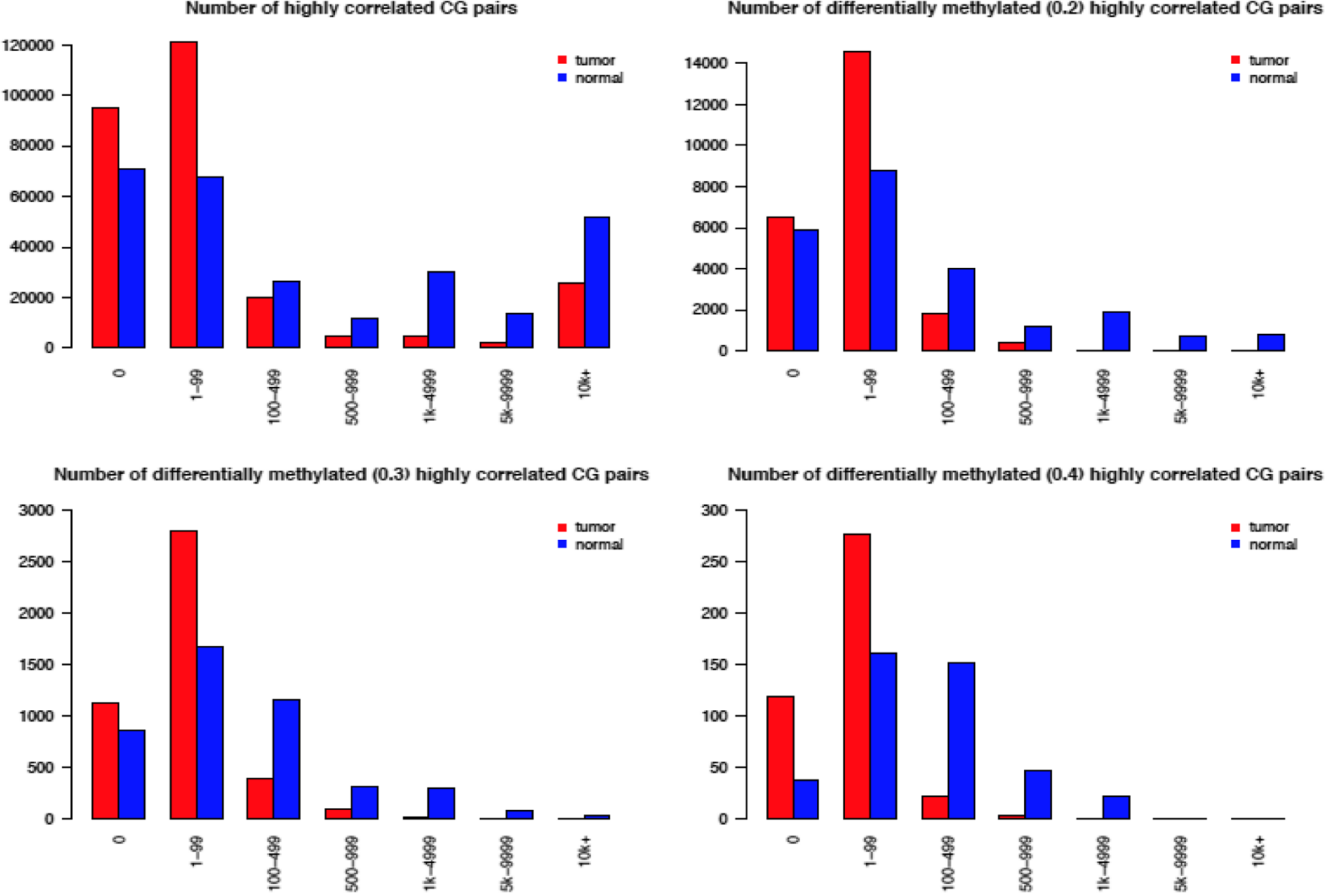
Differentially methylated CG sites for each mean difference value. The above plots are for different mean difference cutoff values: 0 (no DM selection), 0.2, 0.3, and 0.4

In addition to examining the CG sites whose correlations switch from positive to negative and vice versa, we also study the relationship between DM CG sites and the number of CG sites they are highly correlated with, see Supplemental Table 3 in the Additional file 1 and Fig. 5. Supplemental Table 3 shows that among all the 272,990 CG sites, 23,361 (8.56%) are DM. We see that being DM is somewhat associated with the pattern of the number of high correlations. For example, for Tumor (0.2) (that is, the group with 0.2 as the mean difference cutoff value), if being DM is independent from the number of correlations, we would expect each category in the Tumor (0.2) row to be about 8.56% of their respective categories in the Tumor (all) row. However, some categories (e.g., 5 k-9999 and 10 k+) are much lower than expected.

To view the patterns in Supplemental Table 3 in the Additional file 1 clearly, we plot the data presented in this table in Fig. 5. For each of the mean-difference values (0.2, 0.3, 0.4), we plot the number of normal and tumor correlations for each of the intervals in Supplemental Table 3. The number of tumor correlations seems to spike in the 1-99 interval for all plots, and the number drastically drops for the intervals beyond 1-99. This change is not that apparent in the normal data. When comparing the tumor data with the normal data in the 1-99 interval, we also see the tumor data and normal data have the largest percentage difference. For example, in the 1-99 category, that is 62.26% (tumor) vs. 37.51% (normal) when using DM sites selected without a cutoff, as shown in Supplemental Table 3 in the Additional file 1. To compare the numbers of highly correlated sites between the normal and tumor datasets as shown in Fig. 5, we conduct a Wilcox rank sum test using the absolute difference between the normal and tumor sites in each bin (0, 1-99, 100-499, 500-999, 1000-4999, 5000-9999, 10,000+). We get significant test results with small *p*-values: 0.0078 (for Fig. 5 top left plot), 0.0111 (for Fig. 5 top right plot), 0.0078 (for Fig. 5 bottom left plot), and 0.02953 (for Fig. 5 bottom right plot).

### Locations of CG sites

The locations of CG sites are important. Next, we conduct analysis on the locations of different sets of CG sites. There are six location categories: Open_Sea, Island, N_Shelf, N_Shore, S_Shelf, and S_Shore. These correspond to not being associated with a CpG island (i.e., Open_Sea), being on a CpG island, being on a north shelf, being on a north shore, being on a south shelf, and being on a south shore of a CpG island respectively. As for the locations of the original 476,947 CG sites and our selected 272,990 sites, their distributions are shown in Table 7 columns 2 and 3. As we see, the largest category is Open_Sea (about 35%), followed by CpG island (about 30%). North and South regions are around the same, with shore being about 2.5 times the value of shelf. We also conduct this analysis on the highly changing CG site datasets (both.direction, uniq.pos2neg, uniq.neg2pos) as shown in Table 7 columns 4-6. The majority of highly changing sites are mainly in the Open_Sea (40.3 - 55%) and Islands (11.6 -26.1%). The Chi-square test shows that there is a significant association between the types of CG sites (both.direction, uniq.neg2pos, uniq.pos2neg) and the location of these CG sites. For example, when comparing the uniq.neg2pos and uniq.pos2neg groups, we can see that the locations of these two groups are different. The uniq.neg2pos has a larger percentage of CG sites in the Open_Sea than the uniq.pos2neg group (55% vs. 40.3%); it has much smaller percentage of CG sites in the Island than the uniq.pos2neg group (11.6% vs. 26.1%). The Chi-squared test of comparing these groups gives a test statistic of 59.17, with a degree freedom = 5 and a *p*-value = 1.804 × 10^− 11^.

**Table 7:** The locations of different sets of CG sites

	All	Filtered	both.direction	uniq.neg2pos	uniq.pos2neg	Normal Top 100	Tumor Top 100
Open_Sea	170,901 (35.8%)	97,551 (35.7%)	183 (47.5%)	582 (55.0%)	176 (40.3%)	4 (4%)	2 (2%)
Island	148,332 (31.1%)	81,789 (30.0%)	76 (19.7%)	123 (11.6%)	114 (26.1%)	60 (60%)	70 (70%)
N_Shelf	23,109 (4.8%)	11,906 (4.4%)	17 (4.4%)	51 (4.8%)	17 (3.9%)	0 (0%)	0 (0%)
N_Shore	58,427 (12.3%)	36,421 (13.3%)	43 (11.2%)	124 (11.7%)	59 (13.5%)	16 (16%)	15 (15%)
S_Shelf	22,788 (4.8%)	11,667 (4.3%)	14 (3.6%)	54 (5.1%)	13 (3.0%)	0 (0%)	1 (1%)
S_Shore	53,390 (11.2%)	33,656 (12.3%)	52 (13.5%)	124 (11.7%)	58 (13.3%)	20 (20%)	12 (12%)
Total	476,947 (100%)	272,990 (100%)	385 (100%)	1058 (100%)	437 (100%)	100 (100%)	100 (100%)

The first column is the location. The second column is for all 476,947 CG sites. The third column is for the 272,990 CG sites selected for our co-methylation analysis. The fourth to sixth column are for three types of highly changing sites. The last two columns are for super-connector CG sites that are highly co-methylated with other sites.

In addition, we also obtain the top 100 super-connector CG sites in both normal and tumor datasets and analyze their locations as shown in Table 7 columns 7 and 8. The majority of these super-connector sites are on islands (60% for normal, 70% for tumor) or shores (36% for normal, 27% for tumor). We then compare the distribution of the top 100 super-connector CG sites’ locations in each dataset with the overall distribution of all 272,990 CG sites. Since some of the cells have 0 CG sites, we compare specific cells with two-sample tests for equality of proportions. For example, doing this for the Open_Sea category between the overall data and normal top 100 super-connector gives a *p*-value of 7.17 × 10^− 11^ (35.7% vs. 4.0%), and for the Island category, it gives a *p*-value of 1.14 × 10^− 10^ (30% vs. 60.0%). Furthermore, the locations of super-connector CG sites are also very different from the locations of highly changing sites. For example, for the Open_Sea region or category, 40.3 - 55% of the highly changing sites are there, but only 2-4% of the super-connector sites are there. As for the Island region, only 11.6-26.1% of the highly changing sites are there, but 60-70% of the super-connector sites are there. In summary, highly changing sites and super-connector sites have significantly different locations from each other and from the locations of the CG sites in the whole Illumina 450K dataset as well.

### Induced network modules

**Fig. 6:**
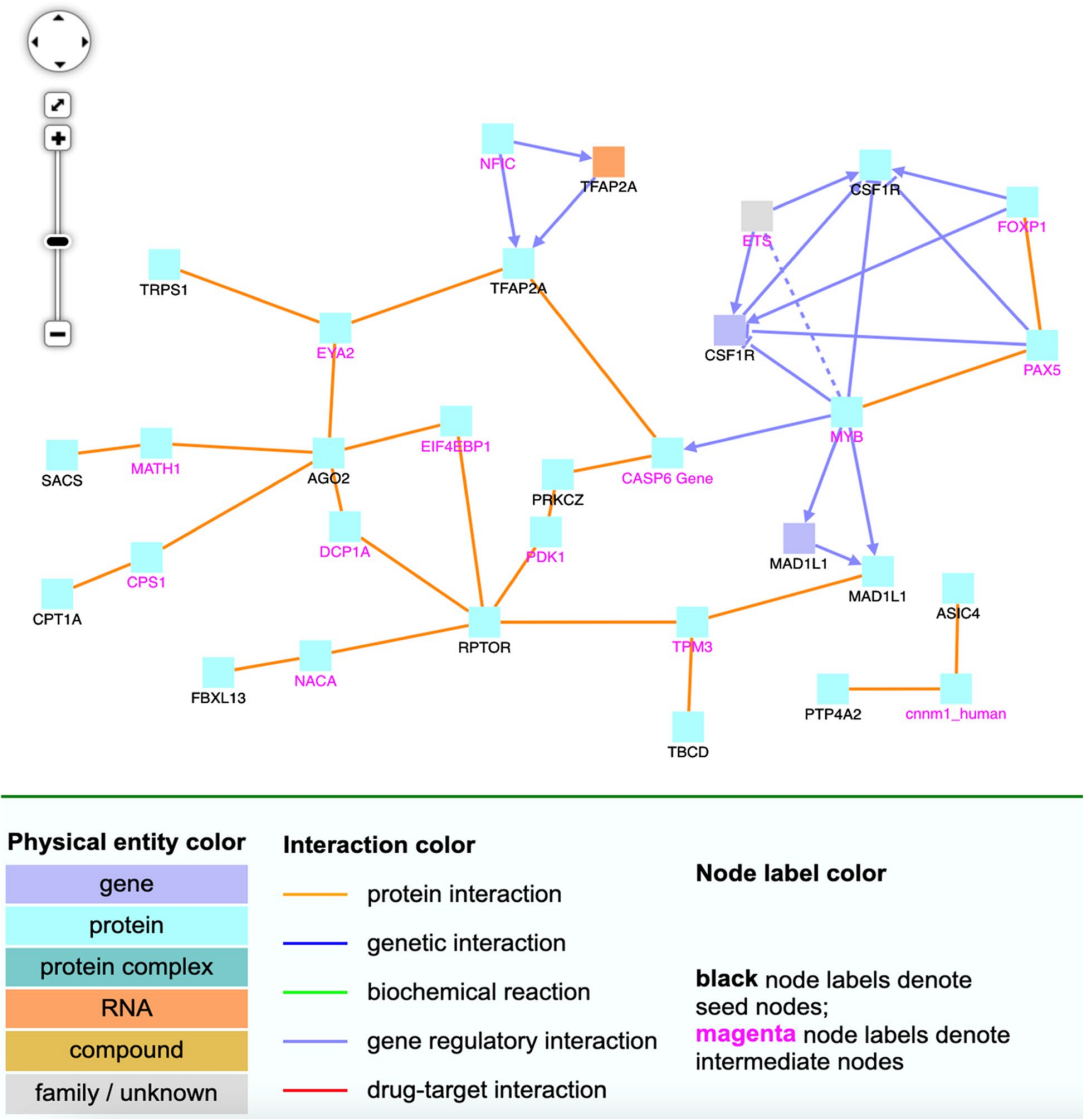
CPDB network modules for genes in the both.direction.DM group. The squares represent genes and the lines represent interactions. Squares with black names are those in our original dataset, while squares with pink names are intermediates added by the CPDB. See the legend at the bottom of this figure for detailed description. Only protein interactions and gene regulation interactions are considered

**Fig. 7:**
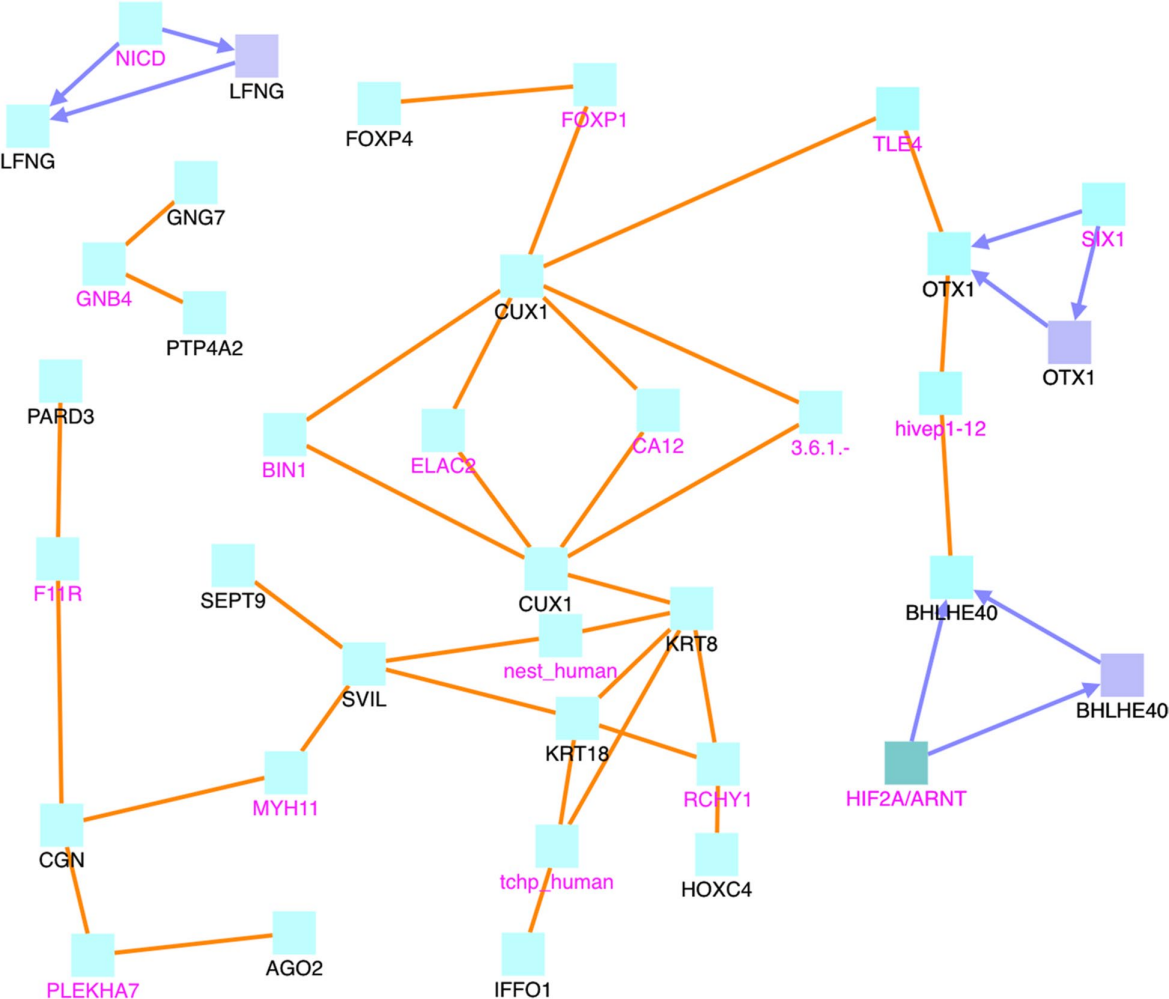
CPDB network modules for genes in the uniq.pos2neg.DM group. The squares represent genes and the lines represent interactions. Squares with black names are those in our original dataset, while squares with pink names are intermediates added by the CPDB. See the legend at the bottom of Fig. 6 for detailed description. Only protein interactions and gene regulation interactions are considered

**Fig. 8:**
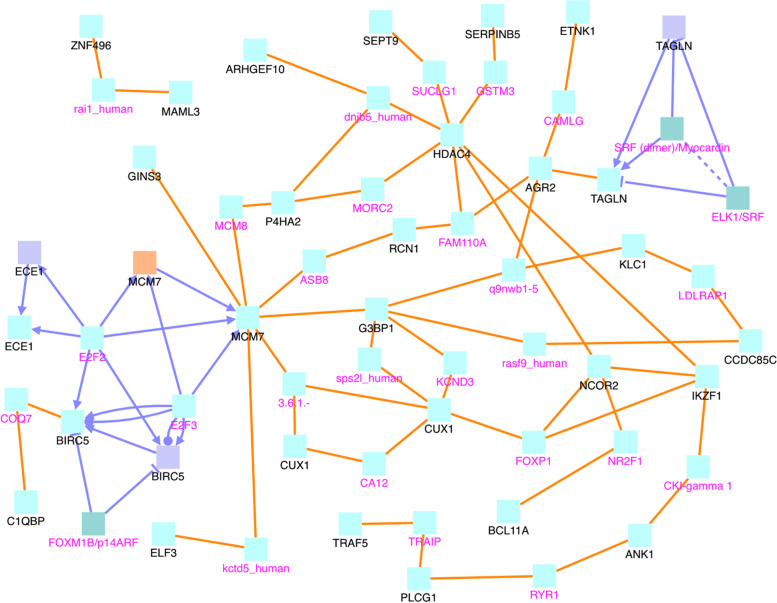
CPDB network modules for genes in the uniq.neg2pos.DM group. The squares represent genes and the lines represent interactions. Squares with black names are those in our original dataset, while squares with pink names are intermediates added by the CPDB. See the legend at the bottom of Fig. 6 for detailed description. Only protein interactions and gene regulation interactions are considered

**Fig. 9:**
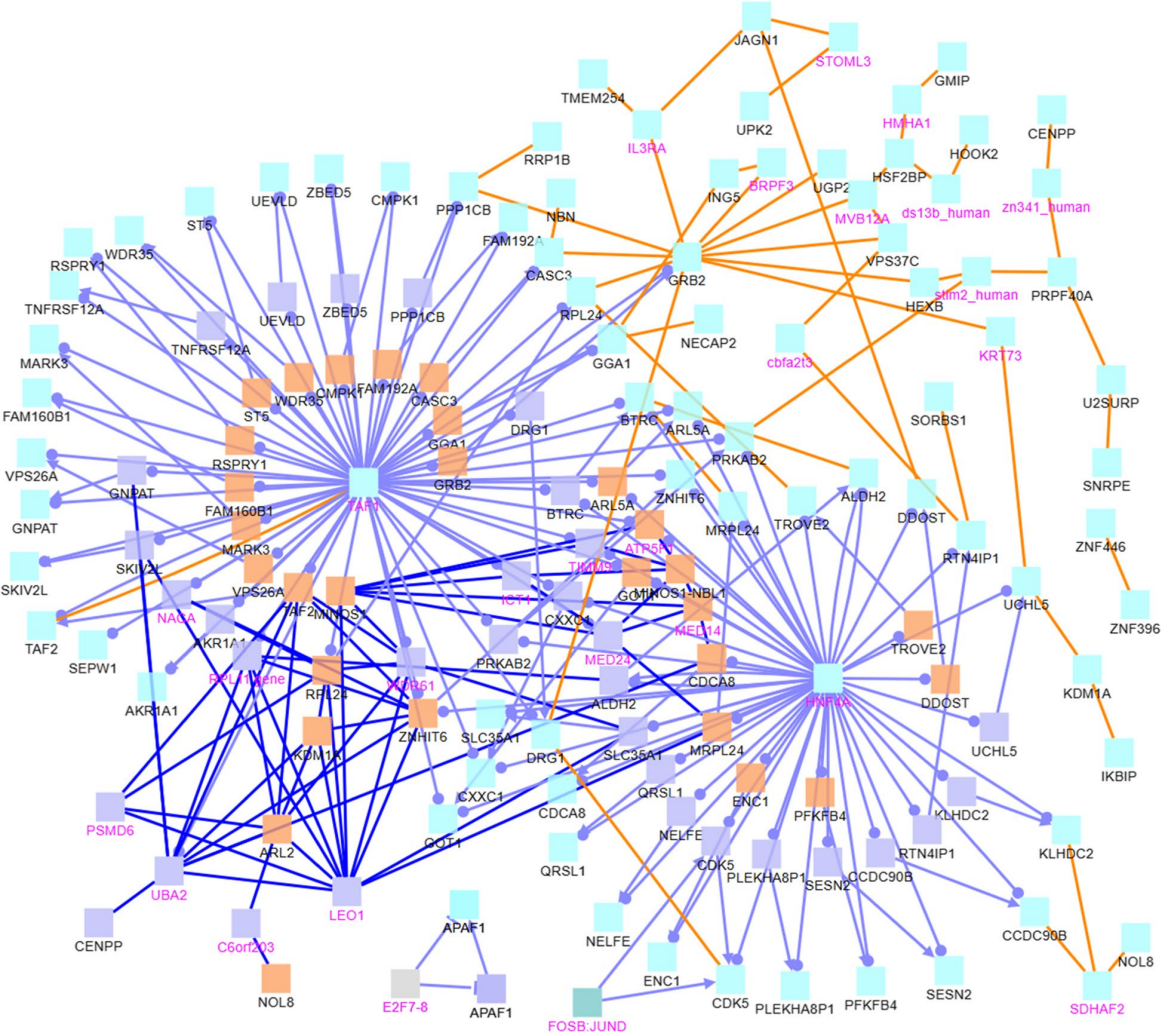
CPDB network modules for genes associated with tumor top 100 highly co-methylated sites. The squares represent genes and the lines represent interactions. Squares with black names are those in our original dataset, while squares with pink names are intermediates added by the CPDB. See the legend at the bottom of Fig. 6 for detailed description. Protein interactions, gene interactions, and gene regulation interactions are considered

**Fig. 10:**
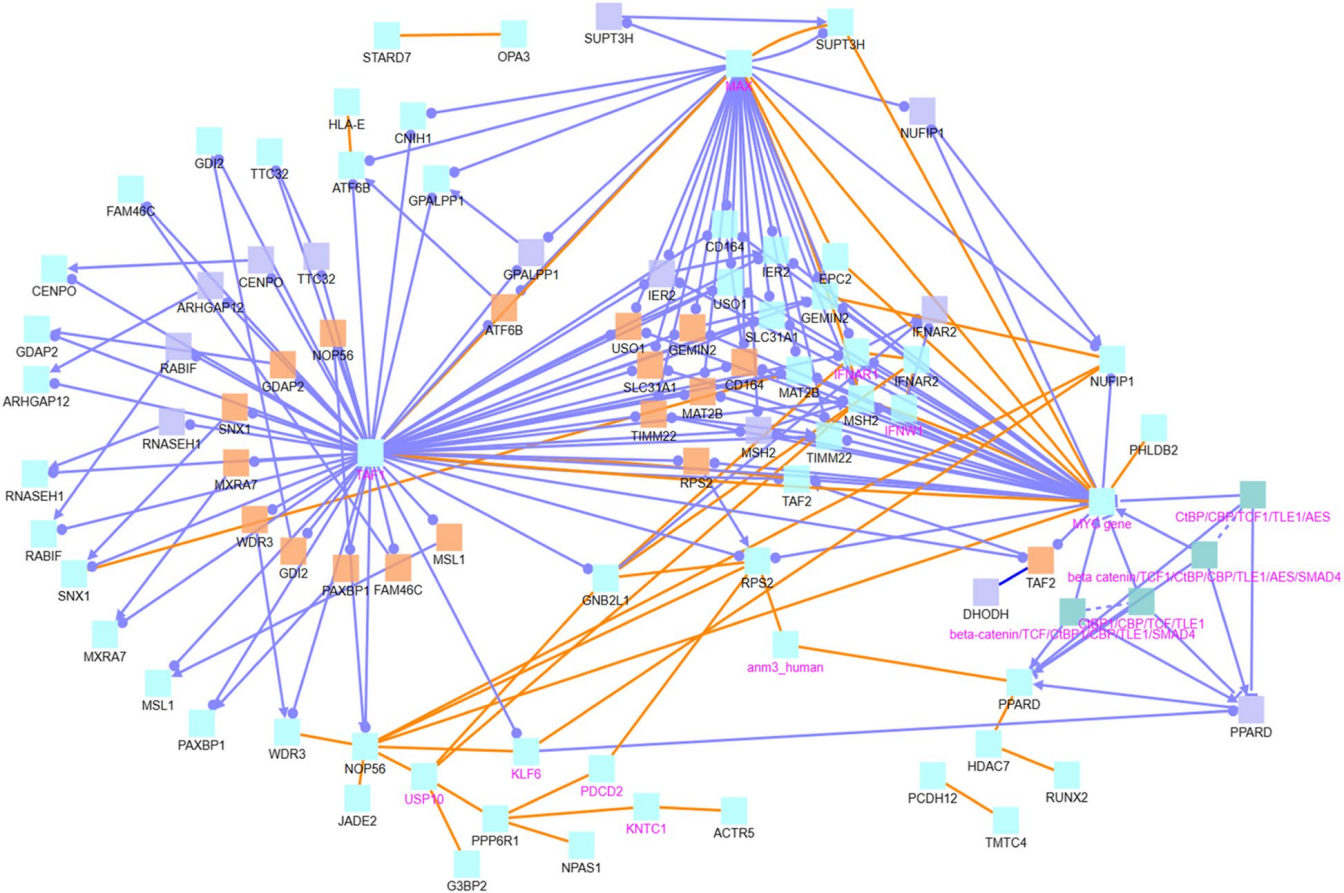
CPDB network modules for genes associated with normal top 100 highly co-methylated sites. The squares represent genes and the lines represent interactions. Squares with black names are those in our original dataset, while squares with pink names are intermediates added by the CPDB. See the legend at the bottom of Fig. 6 for detailed description. Protein interactions, gene interactions, and gene regulation interactions are considered

We study the relationship of the genes associated with CG sites listed in Table 7 using the software ConsensusPathDB (CPDB) [18–20]. We will first discuss the both.direction.DM, uniq.pos2neg.DM, and uniq.neg2pos.DM sets, which are the sets that consist of 50, 47, and 101 CG sites, respectively. The 50 sites in both.direction.DM are mapped to 45 distinct gene symbols, which are then plugged into CPDB, resulting in the network graph in Fig. 6. Note, the legend in the bottom of this figure is also for other CPDB figures (Figs. 7, 8, 9, 10). To avoid redundancy and save space, we do not include this legend in the other figures.

In Fig. 6, the RPTOR, CSF1R, and AGO2 genes are of particular interest. They are hub genes with the most interactions. RPTOR (Regulatory Associated Protein Of MTOR Complex 1) is a protein associated with tuberous sclerosis 1 and tuberous sclerosis. CSF1R is associated with leukoencephalopathy, hereditary diffuse, with spheroids and brain abnormalities, neurodegeneration, and dysosteosclerosis. AGO2 is associated with Lessel-Kreienkamp Syndrome and colorectal cancer. The 47 sites in the uniq.pos2neg.DM set are mapped to 47 gene symbols, see Fig. 7. In this figure, CUX1 and KRT8 are hub genes. Aberrant expression of KRT8 is associated with multiple tumor progression and metastasis; so is CUX1 [21]. Finally, the uniq.neg2pos.DM set consists of 101 CG sites that are mapped to 107 gene symbols, see Fig. 8. In this figure, MCM7, HDAC4, BIRC5, BIRC6, G3BP1, and CUX1 appear to have the most connections. These particular genes are interesting. BIRC6 is involved in regulating apoptosis and p53. Both BIRC5 and BIRC6 have been linked to cancer in other publications [22–28]. MCM7 has been linked to prostate cancer in particular and esophageal squamous cell carcinomas [29, 30]. HDAC4 is also related to cancer [31], as is G3BP1 [32]. As seen in the Fig. 6 image legend, most interactions are protein-protein interactions, with some gene interaction submodules, such as CSF1R, FOXP1, PAX5, ETS, and MYB in Fig. 6. Most entities are protein (cyan), with a few genes (indigo), protein complexes (greenish blue), and RNAs (orange).

We next examine those CG sites that are co-methylated with a very large number of other CG sites. We extract the top 100 CG sites that are co-methylated with the most other CG sites in the tumor data as well as the 100 CG sites that are co-methylated with the most other CG sites in the normal data. There is no overlap between these two sets of CG sites. We then find the genes associated with these sets of CG sites separately and perform the induced network module analysis on these two gene lists, see Figs. 9 and 10.

In the tumor “top100” list (Fig. 9), TAF1 and HNF4A are the main hub genes. In the normal “top100” list (Fig. 10), TAF1, MAX, and MYC genes are the most significant hub genes. Therefore, TAF1 is a hub gene in both the normal and tumor lists, while HNF4A, MAX, and MYC are not. TAF1 (TATA-Box Binding Protein Associated Factor 1) is associated with X-linked intellectual disabilities and X-linked dystonia [33]. TAF1 also phosphorylates p53 on Thr55 [34]. HNF4A (Hepatocyte Nuclear Factor 4 Alpha) is associated with Type 1 Maturity-Onset Diabetes of the Young [35]. HNF4A is also a potential marker for distinguishing between primary gastric cancer and metastatic breast cancer [36]. MYC is a proto-oncogene involved in cell cycle progression and apoptosis; amplification of MYC is observed in numerous human cancers, including breast cancer [37–40]. MAX is the associated factor X of MYC (MAX and MYC together form a protein complex that is a transcriptional activator) and is associated with pheochromocytoma [33]. Note that all of these hub genes are all proteins and also are all intermediate nodes, meaning they are added by the CPDB and are not part of the original input gene lists. It is likely that there are certain genetic or epigenetic changes on such hub genes. These changes may affect the genes in our co-methylation lists, and the majority of their connections are gene regulatory interactions (see light blue lines).

In addition to looking at the highly changing CG sites and highly correlated CG sites, we also investigate the 419 differentially methylated sites in the tumor and normal dataset separately. These 419 sites are selected using *p*-value < 0.05 and mean difference > 0.4. Among these 419 DM sites, we then focus on the CG sites highly correlated with at least 1 other CG site that are only in either normal or tumor data. There are 109 and 29 such CG sites in normal and tumor data respectively. We then conduct the network analysis using the CPDB; see Supplemental Fig. 4 for normal and Supplement Fig. 5 for tumor in the Additional file 1. These two figures show that there are far more connected genes present in the normal dataset, while the genes associated with the tumor dataset do not show many connections. In the normal dataset, the genes PCDHGA5, PCDHGB4, NKX2-1, SKI, RUNX1, sabp4_human, and RARA seem to be the major hub genes. PCDHGA5 is a protein coding gene associated with Wolf-Hirschhorn Syndrome, which is caused by the deletion of a region of chromosome 4. In regards to endometrial cancer, it is also identified as a deregulated gene with different methylation patterns [41]. PCDHGB4 is also a protein coding gene that is identified as a potential passenger gene in a study related to endometrial cancer, and novel mutations of the gene are only found in tumor samples [42]. NKX2-1 is found to be inversely associated with p53 and KRAS mutations [43]. SKI is found to be a negative prognostic marker in the early stages of colorectal cancer [44]. RUNX1 is thought to have a role in breast cancer and endometrial cancer, and reduced levels of it creates an environment which supports tumor growth [45]. When overexpressed, RARA is found to be associated with worse survival rates in colorectal cancer patients [46]. The tumor dataset does not have any notable hub genes. In the normal dataset, the majority of the interactions are protein interactions, which are represented by the orange lines. In the tumor dataset, all of the interactions are protein interactions. Additionally, most of the hub genes exist in the network as proteins.

### Chromosome X

**Fig. 11:**
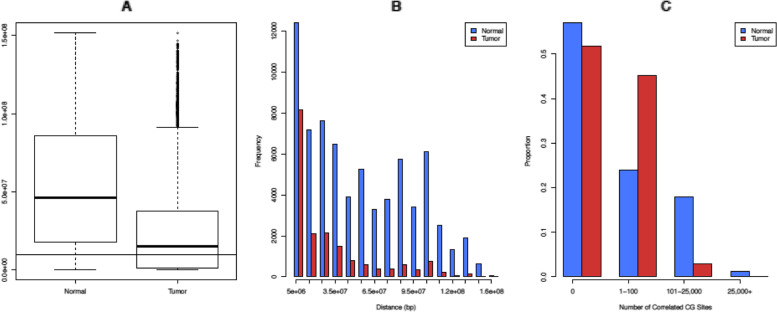
ChrX co-methylation patterns. **A** Boxplots of the distances between correlated pairs on ChrX. **B** Bar plots of the distances between correlated pairs on ChrX. **C** The proportion of ChrX CG sites highly correlated with a certain range of other sites

In the previous section, when we study the overall co-methylation pattern regarding the distance between highly correlated CG sites, we find that ChrX has an outstanding pattern when comparing normal with tumor (see Fig. 11A and Supplemental Fig. 6). That is, the median distance between tumor pairs is much smaller than the median distance between normal pairs, meaning that the tumor pairs are concentrated more closely together than the normal pairs. Due to this finding, we further examine the highly correlated pairs located on ChrX. The ChrX tumor dataset has a much greater percentage of co-methylated sites located very close together than the ChrX normal dataset: 44.7% of tumor pairs are located within 10 million bp of each other compared to only 17.3% of normal pairs, see the horizontal line in Fig. 11A. This is a statistically significant difference with the two-proportion test *p*-value < 2.2 × 10^− 16^. Furthermore, as shown in Table 8, while the maximum absolute distances for the ChrX normal pairs and tumor pairs are very similar, the tumor pairs are concentrated at very close distances to each other, so the median distance between highly correlating sites is about three times larger in the normal data than in the tumor data—that is, 46,352,309 bp (for normal) vs. 15,134,665 bp (for tumor). Note that the ChrX pattern shown in Fig. 11B is very different from the overall pattern of the whole genome as shown in Fig. 2.

**Table 8:** Summary of absolute distances between co-methylated CG pairs on ChrX

	Minimum	1st Quartile	Median	Mean	3rd Quartile	Maximum
ChrX Tumor	2	1,382,456	15,134,665	27,195,461.17	37,411,823	151,495,608
ChrX Normal	2	17,983,484.25	46,352,309	52,772,051.89	85,721,278.75	152,125,211

For each CG site on ChrX, we also examine the number of CG sites that this site is highly correlated with in the tumor and normal data separately, see Fig. 11C. This figure shows that over 50% of ChrX sites do not highly co-methylate with any other CG sites, with the normal data having a slightly higher proportion of such sites than tumor. This proportion is much lower in the full dataset, with around 35% for tumor and an even smaller proportion, about 26%, for normal, as shown in Fig. 1A. However, in both the ChrX data and full data, we observe that there is a considerably higher proportion of tumor sites than normal sites that are highly correlated with 1 to 100 other CG sites, and a higher proportion of normal sites than tumor sites that are highly correlated with more than 100 other sites. Note, the exception to this observation is in the 20,001-30,000 range as illustrated in Fig. 1, with more tumor CG sites than normal CG sites falling into this category, though this is not the case for the ChrX-only data. In summary, we find that the co-methylation patterns in ChrX are very different from those of the autosomes or the whole genome.

**Fig. 12:**
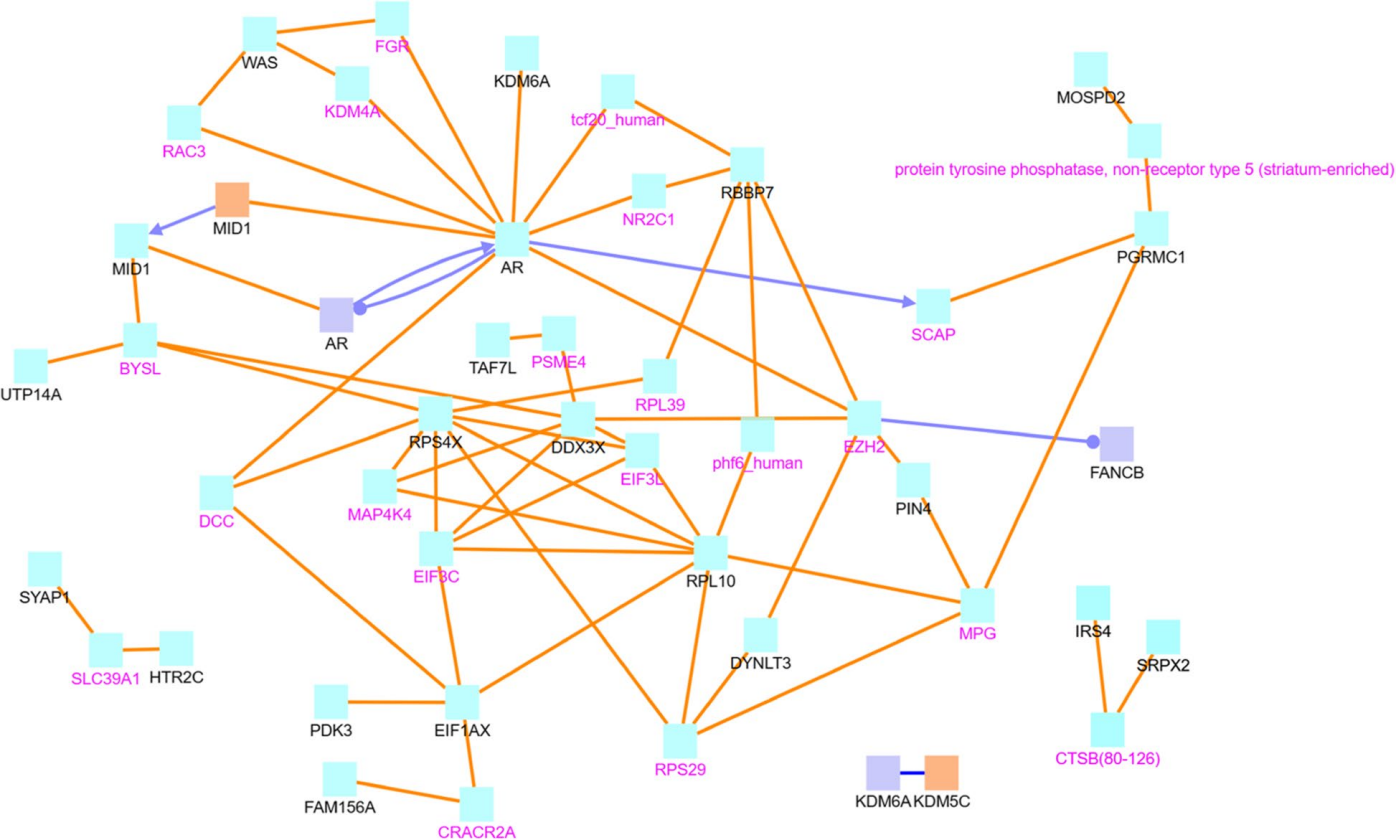
CPDB network modules for genes associated with ChrX tumor data top 100 highly co-methylated sites. The squares represent genes and the lines represent interactions. Squares with black names are those in our original dataset, while squares with pink names are intermediates added by the CPDB. See the legend at the bottom of Fig. 6 for detailed description. Protein interactions, gene interactions, and gene regulation interactions are considered

**Fig. 13:**
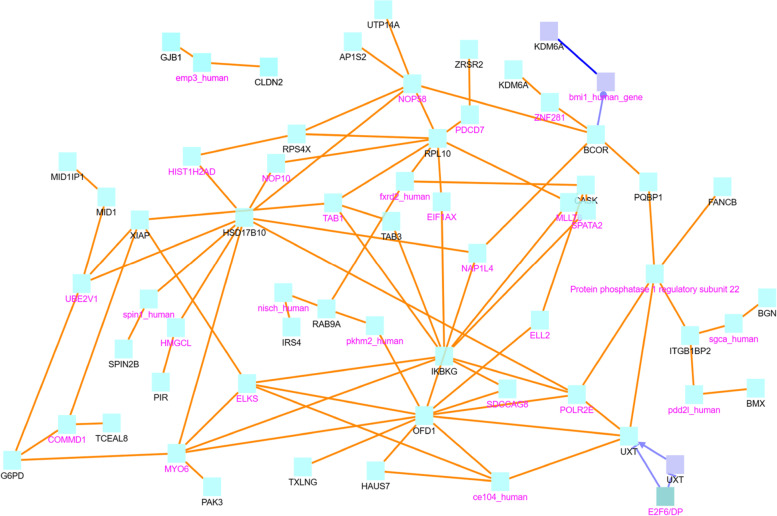
CPDB network modules for genes associated with ChrX normal data top 100 highly co-methylated sites. The squares represent genes and the lines represent interactions. Squares with black names are those in our original dataset, while squares with pink names are intermediates added by the CPDB. See the legend at the bottom of Fig. 6 for detailed description. Protein interactions, gene interactions, and gene regulation interactions are considered

Finally, we also investigate the top 100 ChrX super-connector CG sites that are highly co-methylated with other sites to see their impact on breast cancer development. After creating separate lists of genes associated with these ChrX super-connector CG sites in tumor data and normal data, we perform the CPDB induced network module analysis for these lists (see Figs. 12 and 13). In the tumor gene list, we identify the key hub genes AR, RPL10, and RPS4X in Fig. 12. In the normal gene list, we likewise identify RPL10 along with HSD17B10, OFD1, and IKBKG as key hub genes in Fig. 13.

Next, we demonstrate the findings related to hub genes in tumor data as shown in Fig. 12. AR is the androgen receptor gene and is expressed in the majority of primary breast tumors [47]. Lehmann et al. even identify luminal androgen receptor (LAR) as a subtype of triple negative breast cancer [48]. RPL10 is a protein coding gene associated with X-linked developmental disorders. Interestingly, Fang and Zhang find RPL10 to be a hub gene and potential biomarker for breast cancer [49]. RPS4X is a ribosomal protein that has been studied as a potential prognostic marker for ovarian cancer, with low levels of the gene associated with poor survival [50].

Below are the findings related to hub genes in normal data shown in Fig. 13. HSD17B10 is identified as a key molecule involved in cell proliferation and death [51]. Notably, its expression is enhanced in the presence of fulvestrant, a method of ER+ advanced breast cancer treatment, suggesting an important role of this gene in fulvestrant resistance. IKBKG is significantly upregulated in inflammatory breast cancer (IBC) tumor samples in comparison to regular breast cancer tumor samples (non IBC), and it is more down-regulated in IBC metastases in comparison to that of non IBC [52]. OFD1, a gene underlying oral-facial-digital syndrome 1, controls the length of centrioles [53]. Tang et al. find that a lack of OFD1 at centriolar satellites facilitates cilia formation in transformed breast cancer MCF7 cells, which normally does not contain cilia [54]. This finding suggests that OFD1 depletion at centriolar satellites provides a promising way to promote ciliogenesis in mammalian cells.

### Comparing with other related studies

Next, we compare our study with the two most relevant co-methylation studies. One is for breast cancer [11] and another is for colon cancer [55], see Table 9. The first several rows of this table show how the three studies are similar or different regarding tissue/cancer type, data size, analysis unit, tumor/normal sample used and so on. One key similarity of these three studies is that we all analyze the relationship between co-methylation and genetic distance. We all find a weak negative correlation between the co-methylation and genomic distance for CG pairs on the same chromosome. As for the tumor and normal samples, Akulenko and Helms do not separate them in their analysis, so no comparison is done [11]. Mallona et al. compare tumor and normal data and find that they have different co-methylation patterns although their analysis and our study are conducted from different perspectives and with different approaches [55]. In addition, we conduct analyses in five additional aspects that Akulenko and Helms do not do: negative correlation, number of high correlation partners (or co-methylation degree), ChrX co-methylation, highly changing sites, and relationships with DM sites. Furthermore, Akulenko and Helms report 74 out of 187 co-methylated gene pairs on the same chromosome [11]; we report 45.2 million (i.e., 5.7%) CG pairs for normal and 17.5 million (i.e., 5.87%) CG pairs for tumor on the same chromosome. Finally, Akulenko and Helms’ study is based on the Illumina 27K data, which is about 10 times less than the data we used. Therefore, our findings show a bigger and clearer picture of co-methylation patterns as more CG sites are used.

**Table 9:** Comparing three co-methylation studies

	2013 study by Akulenko and Helms	2020 study by Mallona et al.	Our study
Tissue/Cancer	Breast cancer	Colon cancer	Breast cancer
Data type	Illumina 27K (27500)	Illumina 450K (485577)	Illumina 450K (485577)
CGs or genes used	13,133 genes	~ 300,000 CG sites	272,990 CG sites
Analysis unit	Gene	CG (or probe)	CG (or probe)
Sample size	317 tumor and 27 adjacent normal	(1) 90 tumor and 90 adjacent normal (2) 256 tumor and 38 adjacent normal	53 tumor and 53 adjacent normal
Tumor vs. normal	Combined	Separated & compared	Separated & compared
Relationship with genomic distance	Yes	Yes	Yes
Gene or CG pairs on the same chromosome	74/187 gene pairs	No specific result	45.2 million CG pairs for normal; 17.5 million CG pairs for tumor
Negative correlation	No	Yes	Yes
Number of high correlation partners	No	Yes	Yes
ChrX co-methylation	No	No	Yes
Highly changing sites	No	No	Yes
Relationship with DM sites	No	No	Yes

## Discussion

Our study provides thorough analyses of breast cancer co-methylation patterns. It is different from some available publications. First, we thoroughly compare tumors with matched normal samples, while some other studies often combine those samples together. Second, we not only just look at which two CG sites are co-methylated, but also zoom in to check the genome-wide co-methylation in detail. For example, we address the following questions: for each CG site, how many sites are highly correlated with it; what is the pattern; and how many pairs are positively or negatively correlated. Third, our analyses are conducted at the CG site level, but others’ analyses are conducted at the gene level by taking the average methylation of all CG sites associated with a gene, which can lead to biased results. This bias is due to the fact that CG sites associated with a single gene (especially a long gene) may have very different methylation levels ranging from 0 to 1. Our fine analyses at the CG site level help us identify the highly changing CG sites that no other publications find, which we will explain in the next paragraph. In summary, to our best knowledge, our study is the first one that thoroughly investigates breast cancer co-methylation patterns from different perspectives that are not considered by previous studies.

The idea for the 8 × 8 CG pairs matrix comes from the paper by Tang et al. [17]. This paper features two similar matrices comparing Rheumatoid Arthritis and Parkinson’s disease though they compare gene pairs instead of CG pairs. The authors map 485,577 CG sites or probes in the Illumina Human Methylation 450K data to 21,225 genes. If there are multiple CG sites associated with a gene, they calculate the average beta values of those sites as the methylation value of the gene. In our study, we choose to thoroughly analyze the co-methylation patterns at the CG site level as the multiple CG sites associated with the same gene may have totally different methylation levels, especially those CG sites that are far away from each other. For very long genes that have multiple sites, this difference is likely very large. In the whole genome, about 70% of genes’ lengths are larger than 10,000 bases pairs, and 50% of genes whose lengths are larger than 21,000 base pairs. Using the average methylation for all CG sites associated with one gene will likely produce a biased methylation signal for those long genes. Therefore, we analyze the co-methylation patterns at the CG site level to see a clearer picture and get more accurate results. In particular, as for identifying highly changing CG sites, we borrow the idea from Tang et al. [17]. In their Tables 1 and 2, there are many 0s in the left bottom and right top corners. That is, their analyses do not identify highly changing sites. Although this difference could be due to the fact that we study different tissues, we think the difference may be more likely due to the fact that their analysis is done at the gene level, while our analysis is conducted at the CG site level.

From our work on smaller subsets of the Illumina 450K data, we know that data filtering can have an impact on the results of the analysis as the number of CG sites changes with different filtering methods. We consider a number of different filtering criteria, including standard deviation (stdev) and IQR bounds and outlier counts. The results are summarized in Supplemental Table 4 in the Additional file 1. As we see, the stdev≥0.025 (the third column) is the only variance filtering criterion that leaves more than 50% of the data for both the tumor and the normal data. In all of the variance filtering criteria, a significantly greater percentage of the CG sites in the normal data are filtered out, which fits with the greater homogeneity of the normal epigenome. However, filtering by outliers and filtering by missing values, i.e., NAs (see columns 7-9), removes about the same number of CG sites in both datasets. Removing all the sites with more than 11 NAs leaves about 82% of sites. Filtering outliers leaves about 78% of the data when using coef = 2 and 83-85% of the data when using coef = 3. After careful consideration, we decide to use the filtering criteria in the Methods section to ensure that there is enough variation among methylation signals and that we can also have a reasonable number of CG sites for analysis.

There are a few interesting findings about negative correlations in our analysis. There are generally fewer negative correlations than positive correlations and generally fewer negative correlations in tumor samples than in normal samples. Despite this, there are still a small number of CG sites that only have negative correlations in the normal and tumor datasets. A couple of previous studies discuss negative correlations in methylation, though not in as much detail as our study. One such study is the paper by Ding et al. on co-occurrence and mutual exclusivity [16]. This paper uses co-occurrence for positive co-methylation and mutual exclusivity for negative co-methylation. The authors define the beta value > 0.3 as methylation and < 0.3 as unmethylation. They also use the average beta values in the promoter region of a gene instead of the value for each CG site. Another study is the paper by Mallona et al. [55], who study negative correlation (called anti-methylation by the authors) at the CG site level but focus more on positive correlations. To the best of our knowledge, our research work is the first study that thoroughly investigates negative co-methylation patterns by comparing tumor and normal samples.

In another breast cancer methylation study, Sun et al. show that about 60% of CG sites are DM when using the paired t-test (*p*-value < 0.05), with the proportion for ChrX slightly larger than other chromosomes (see Fig. 5 C of Sun et al. 2015) [56]. Without any multiple-test correction, it is likely that there are a large number of false positive sites. We find that after using both *p* < 0.05 and the mean difference > 0.2 to remove some false positive sites, the average DM% of all autosomes is 8.62%, but ChrX is only 2.22%, as shown in Supplemental Table 2 in the Additional file 1. In addition, we also find that among all DM sites on almost all chromosomes, there is a greater percentage of hypermethylated sites than hypomethylated sites. The percentage differences on some chromosomes, such as Chr4 and Chr5, are even around 30-40%. Only ChrX and Chr8 have a larger percentage of hypomethylated DM sites. However, our DM analysis is only based on 273K out of the total 28 ~ 29 million CG sites in the whole genome (that is, only about 1% of them). It is worth conducting more research using the whole genome bisulfite sequencing data.

In this paper, we find that some ChrX co-methylation patterns are different from the ones in the other chromosomes. We show that on ChrX, co-methylated tumor CG pairs tend to be located much closer together than co-methylated normal pairs, with a significantly larger number of tumor CG pairs located within 10 million bp of each other than normal pairs. In the full dataset and in the other individual chromosomes, the distribution of the distances does not change as much from normal to tumor. We also observe a larger percentage of ChrX sites that do not co-methylate with any other sites than the percentage of such sites in the full dataset. These results add to the existing literature regarding the importance of epigenetic changes in ChrX. As discussed previously, Sun et al. also analyze Illumina 450K methylation data and a similar filtered number (9653) of ChrX CG sites [56]. They find that DM patterns for ChrX are different from the other chromosomes. They also find cell lines without ChrX loss to be more active in gene expression [56]. Chaligné et al. demonstrate the epigenetic instability of the inactive X chromosome (Xi) and propose that the Xi could be used as an epigenetic biomarker at the molecular and cytological levels in cancer [57]. Thakur et al. investigate the role of X-linked genes in breast cancer and find that these genes are important for maintaining chromatin structure, chromosome segregation, and translational control. They suggest that changes in the expression of X-linked genes can lead to increased genetic instability and tumor cell growth [58]. In another publication, Thakur et al. show that high expression of X-linked gene RbAp46 is likely to play a role in the development or progression of human breast cancer [59]. Furthermore, the most recent publication by Cui et al show that the simultaneous activation and repression of the X-linked endogenous gene FOXP3 may provide a potential therapeutic option for female breast cancer [60]. Based on our and others’ results, ChrX should be studied more thoroughly in regard to its epigenetic patterns in relation to breast cancer.

As we explain in the Introduction section, we are studying BS co-methylation. For the highly co-methylated CG sites in the same chromosome, their distances are much larger than the distances reported in our previous WS co-methylation studies [13, 14]. One main reason is that our WS co-methylation studies are conducted using DNA methylation sequencing datasets that have better resolutions than the Illumina 450K data. That is, many more CG sites’ methylation signals are available and the distance between two consecutive CG sites can be less than 10 bps in the methylation sequencing data. However, for the Illumina 450K data, the median distance between any two consecutive CG sites on most chromosomes is about 400 ~ 700 bases. The average distance is much larger than the median distance as the distance distribution is very right skewed. These large median and mean distances explain why we find a relatively large distance for co-methylated CG sites that are on the same chromosome.

Co-methylation patterns may be closely associated with breast cancer development. This association could be due to the complex regulatory role that methylation plays in gene expression. That is, methylation patterns of different genes can have both positive and negative correlations with gene expressions as shown in our previous study [61]. Through co-methylation, genes may work as a network (i.e., “in a team”), having an obvious or subtle impact on other individual genes or networks of multiple genes. Many of the hub genes that we identified with the network analysis (Figs. 7, 8, 9, 10) are involved in cell growth, tumor suppression, or have been otherwise linked to cancer development. The genetic or epigenetic changes of such hub genes may play an important regulatory role on other genes in their networks as their connections are mainly gene regulatory interactions (see the light blue lines in Figs. 9 and 10).

Breast cancer patients often have different positive (+) or negative (−) statuses for the Estrogen Receptor (ER), Progesterone Receptor (PR), Human Epidermal Growth Factor receptor 2 (HER2), which are used to define breast cancer subtypes. Breast cancer diagnosis and treatment methods are related to these hormone receptor statuses and tumor subtypes. There can be some variations for the definition of tumor subtypes [62, 63]. For the sake of convenience, we use the following definition based on the available ER, PR, and HER2 information in our data: Luminal A (ER+ and/or PR+, HER2-), Luminal B (ER+ and/or PR+, HER2+), triple-negative (ER- and PR- and HER2-), and HER2-enriched (ER- and PR- and HER2+). We find that the numbers of different tumor subtypes are 23 Luminal A, 5 Luminal B, 4 Triple Negative, and 2 HER2-enriched. 19 (out of 53) samples’ subtypes cannot be determined due to the lack of information or data. Because the sample sizes for the Triple Negative (only 4) and HER2-enriched (only 2) are too small, and more than 1/3 of samples’ subtypes are not available, the methylation analysis based on the tumor subtypes of our data will not represent the population patterns. Therefore, we do not show any methylation analysis based on tumor subtypes.

DNA methylation and histone acetylation are closely related to each other [64]. On the one hand, histone modifications (acetylation and methylation) and nucleosome positioning can determine DNA methylation patterns [65]. On the other hand, DNA methylation also recruits methyl-CpG binding proteins that affect chromatin structure through the activity of histone deacetylase complexes (HDACs) [66]. With this mechanical dependence, DNA methylation and histone acetylation play key roles in regulating gene expression, and thus affecting cancer development [67, 68]. For example, Lee et al show that the acetylation of the oncogenic transcription factor STAT3 is crucial for the promoter region methylation of several tumor suppressor genes [69]. This process partially explains abnormal gene silencing in cancer. Lee et al also show that the reduction of acetylated STAT3 leads to the demethylation and activation of the estrogen receptor-α gene in triple-negative breast cancer cells. This STAT3 gene is just one typical example. The complex relationship between DNA methylation and acetylation remains to be investigated for other genes in the whole genome. Further studies will provide valuable information for cancer diagnosis and treatment.

## Conclusion

In this article, we have analyzed Illumina 450K methylation data to study co-methylation patterns in breast cancer. From these analyses, we find that the majority of highly correlated CG pairs are located at different chromosomes. Only a small percentage (5 - 6%) of them are on the same chromosome. The highly co-methylated CG sites that are on the same chromosome tend to be located relatively close to each other. In general, the normal dataset has more highly correlated pairs than the tumor dataset, but overall distributions (i.e., the distance-histogram shapes) appear to be the same. We also find some pairs of CG sites whose correlations change highly between normal and tumor samples. The CG sites involved in these pairs tend to possess characteristics different from the other sites. For example, they are more likely to be differentially methylated. We also find that normal samples have a larger proportion of negatively correlated CG pairs than tumor samples. The super-connector CG sites in tumor and normal samples function differently. The locations of highly changing CG sites and super-connector CG sites are significantly different from each other. They are also different from the overall distribution of all CG sites in the Illumina 450K data. The network analyses for genes with specific co-methylation patterns also show that tumor and normal samples have different gene/protein interactions. These genes in tumor and normal samples are associated with different genetic pathways and networks. As stated before, studying co-methylation patterns by comparing tumor samples with matched normal samples can help establish relationships between different genes. It can also serve as an indicator to discover new genes that should be monitored more carefully for breast cancer. Understanding the specific co-methylation patterns of genes involved in certain diseases can ultimately lead to better treatment plans for the patients affected by the disease. These genes can also be used in patient stratification and gene therapy.

## Supplementary Information

**Additional file 1.** Supplemental information (figures and tables). Supplemental figures and supplemental tables.

## Data Availability

The original or raw datasets supporting the conclusions of this article are publicly available in the TCGA repository, https://portal.gdc.cancer.gov/. The datasets supporting the conclusions of this article are included within the article and its additional file.

## Abbreviations

CG: Cytosine-Guanine
Chr: Chromosome
CPDB: Consensus Path Database
TCGA: The Cancer Genome Atlas
IQR: Interquartile range
WS: Within Sample
BS: Between Sample
