# Supplementary material for: Thorough statistical analyses of breast cancer co-methylation patterns
Source: BMC Genom Data. 2022 Apr 15;23:29. doi: 10.1186/s12863-022-01046-w (PMC9011975; doi:10.1186/s12863-022-01046-w)
Supplement: Supplementary file 1 — Additional file 1. Supplemental information (figures and tables). Supplemental figures and supplemental tables. [file 12863_2022_1046_MOESM1_ESM.docx]

**Additional File 1**

**Thorough statistical analyses of breast cancer co-methylation patterns**

**Shuying Sun^1†^, Jael Dammann^2^, Pierce Lai^3^, Christine Tian^4^**

1. Department of Mathematics, Texas State University, San Marcos, Texas, USA, ssun@txstate.edu
2. St. Stephen’s Episcopal School, Austin, Texas, USA, jael.j.dammann@gmail.com
3. Massachusetts Institute of Technology, Cambridge, Massachusetts, USA,

pierce.lai37@gmail.com

1. Liberal Arts and Science Academy, Austin, Texas, USA, christinetian01@gmail.com

†. Corresponding Author

**This additional file 1 includes a few supplemental figures and tables of the above paper.**

**Supplemental Figures**


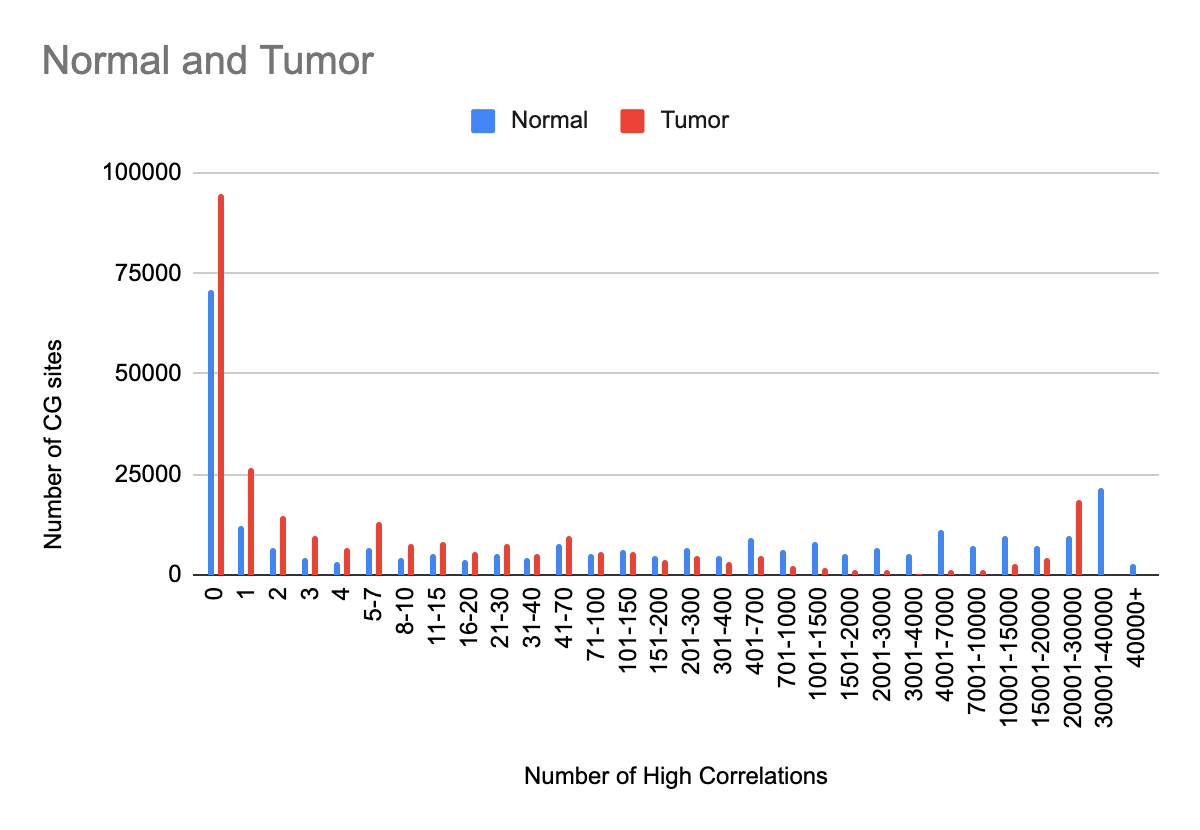


**Supplemental Figure 1.** Number of CG sites that each CG is highly correlated with in tumor and normal data. This figure is a zoomed-in version of Figure 1 with a detailed range of highly correlated partner numbers.


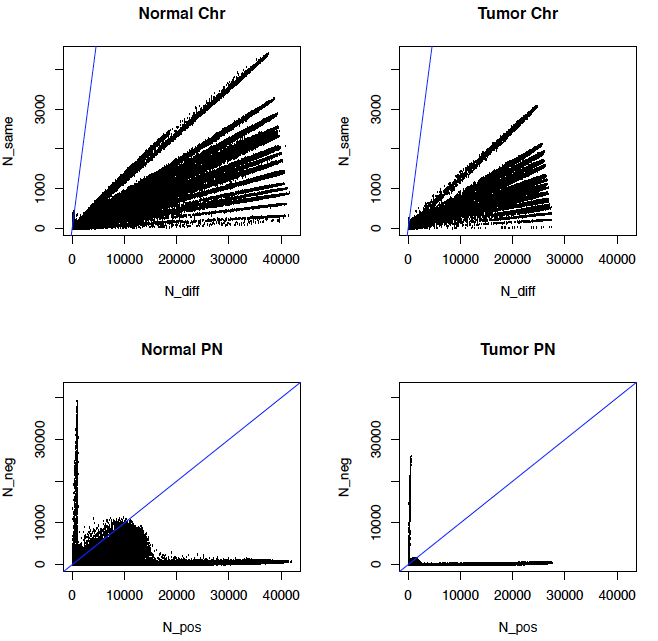


**Supplemental Figure 2**. Scatterplots comparing correlation patterns in normal and tumor data. The top two are scatterplots of the number of CG pairs on the same vs. different chromosomes for each CG site. N_diff means the number of highly correlated CG partners on different chromosomes, and N_same means the number of highly correlated CG partners on the same chromosome. The bottom two are scatterplots of the number of positively vs. negatively correlated pairs for each CG site. N_neg means the number of CG sites that each CG site is negatively correlated with, and N_pos means the number of CG site that each CG site is positively correlated with.


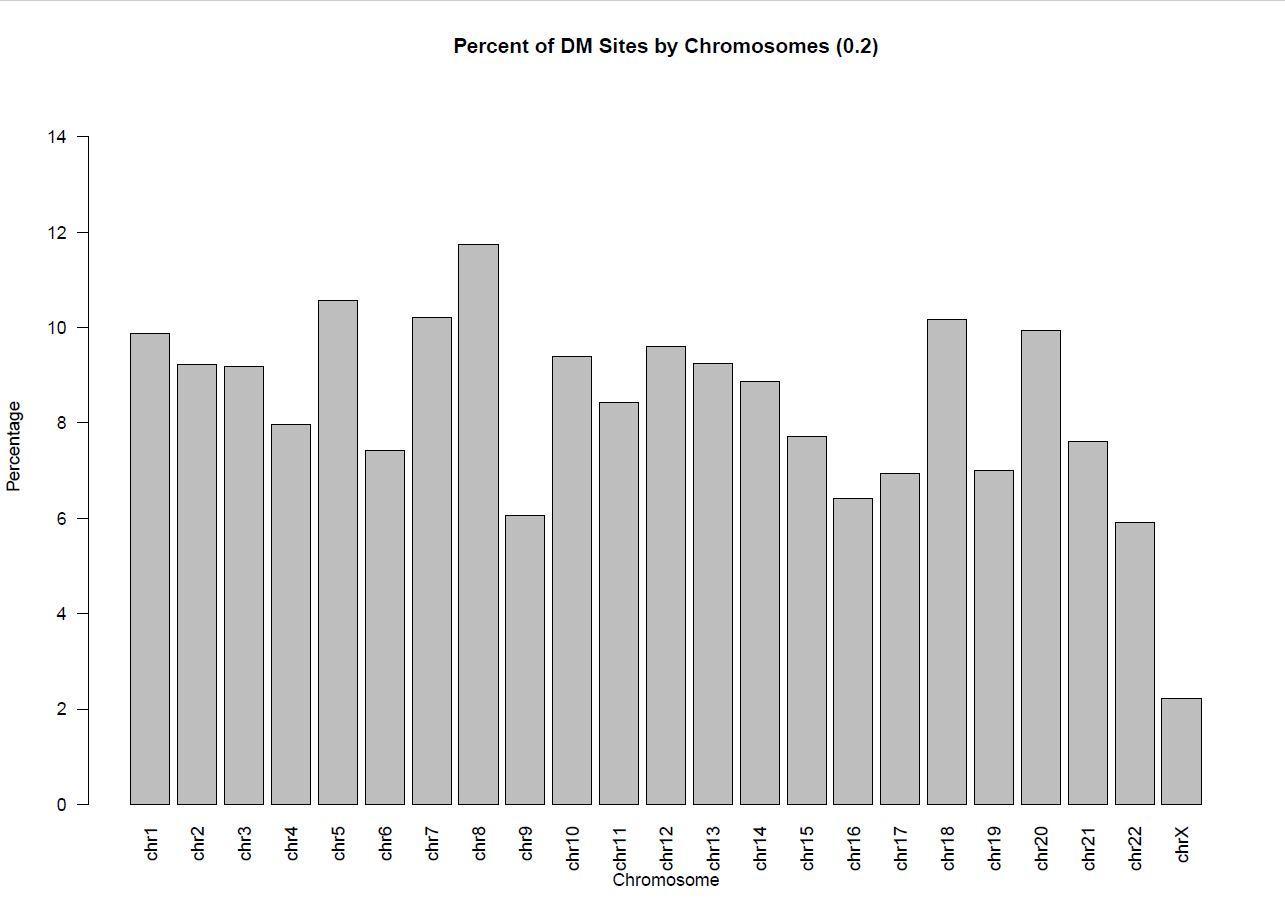


**Supplemental Figure 3**. The percentage of CG sites that are differentially methylated by chromosome. The CG sites are selected based on p-value <0.05 and absolute mean difference >0.2


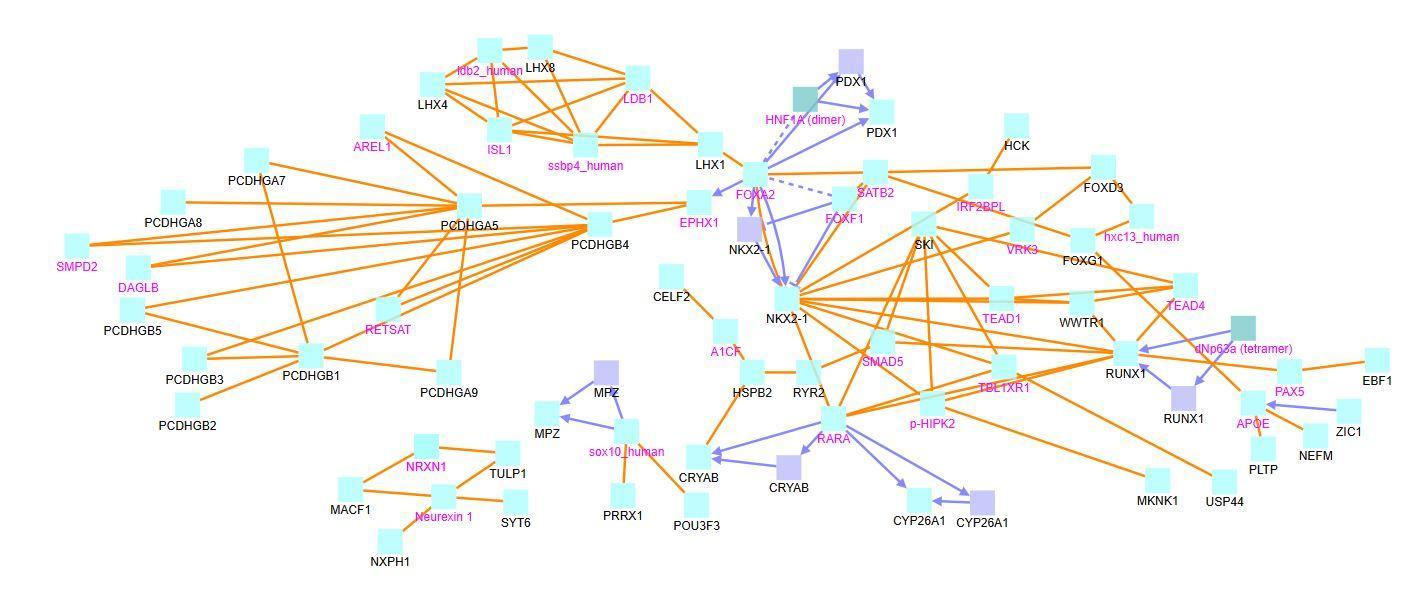


**Supplemental Figure 4**: CPDB induced network modules for genes associated with differentially methylated CG sites that are correlated with ≥1 CG site. The related 109 CG sites are only present in the normal dataset. The squares represent genes, and the lines represent interactions. Squares with black names are those in our original dataset, while squares with pink names are intermediates added by the CPDB. See the legend at the bottom of Figure 6 for detailed description.


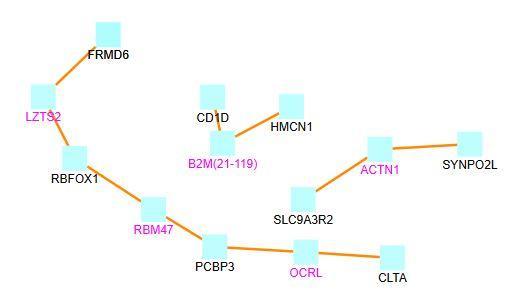


**Supplemental Figure 5.** CPDB induced network modules for genes associated with differentially methylated CG sites that are correlated with ≥1 CG site. The related 29 CG sites are only present in the tumor dataset. The squares represent genes, and the lines represent interactions. Squares with black names are those in our original dataset, while squares with pink names are intermediates added by the CPDB. See the legend at the bottom of Figure 6 for detailed description.

| 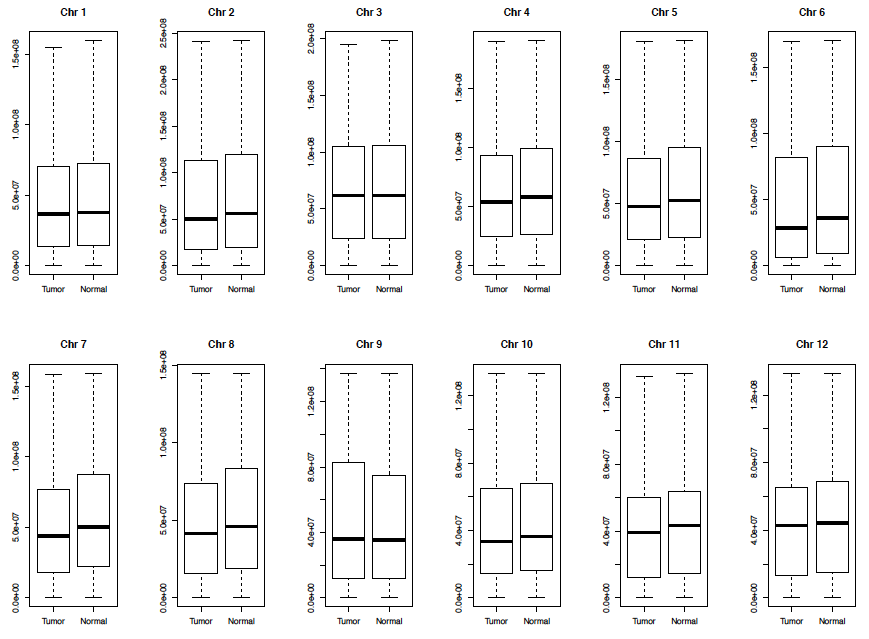  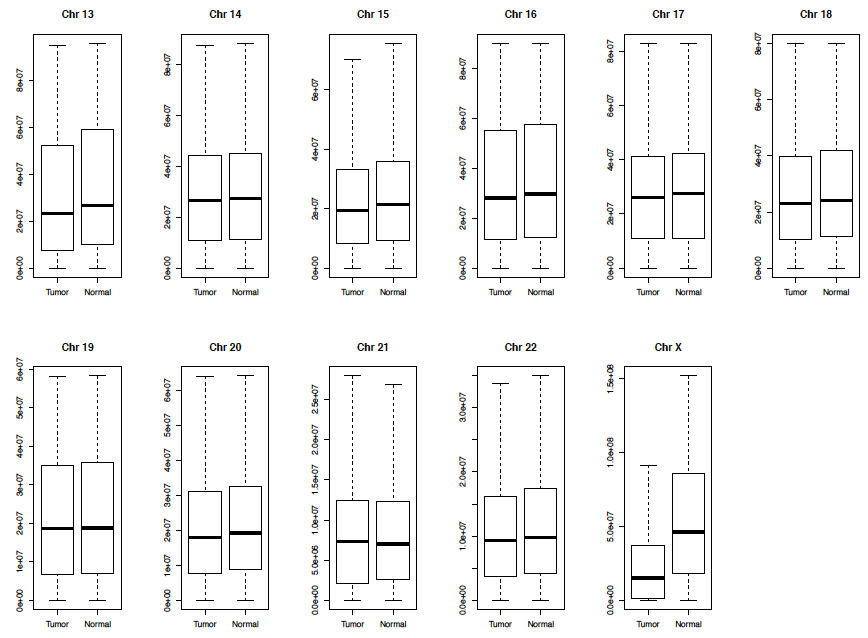 |
| --- |

**Supplemental Figure 6.** Co-methylation pattern by chromosome. Each side by side boxplot graph is for one chromosome and it is to compare the distances between correlated sites in tumor and normal data.

**Supplemental Tables**

**Supplemental Table** 1. T-test results for CG-pair distances by chromosome. N.mean and N.Median are the normal data mean and median; T.mean and T.median are the tumor data mean and median.

|  | N.mean | T.mean | N.median | T.median | p-value |
| --- | --- | --- | --- | --- | --- |
| chr1 | 89044165.86 | 86463826.81 | 76996201.5 | 75174603 | 0 |
| chr2 | 85598064.83 | 81800688.86 | 74340647 | 70390551 | 0 |
| chr3 | 68113324.15 | 67733706.93 | 61776738 | 61678382.5 | 8.56E-12 |
| chr4 | 65942052.8 | 61637849.38 | 57906493 | 53670876 | 0 |
| chr5 | 62035238.07 | 56817084.35 | 52595113 | 47743043 | 0 |
| chr6 | 51275480.37 | 46420480.98 | 36106184.5 | 28501942 | 0 |
| chr7 | 57106126.45 | 50835900.32 | 50173528.5 | 43854931 | 0 |
| chr8 | 53021619.45 | 46924550.67 | 45894011 | 41353911 | 0 |
| chr9 | 45325181.51 | 46965414.37 | 35431251.5 | 35983876.5 | 2.25E-63 |
| chr10 | 44222764.46 | 41805610.06 | 36648311.5 | 33516146 | 0 |
| chr11 | 44361322.06 | 40665670.57 | 43386598.5 | 39130393 | 0 |
| chr12 | 46424364.37 | 44010985.63 | 44338595 | 42708665 | 0 |
| chr13 | 34743685.02 | 30883916.18 | 26775196 | 23278813 | 0 |
| chr14 | 30426116.68 | 29560489.39 | 27405169 | 26724110.5 | 4.23E-99 |
| chr15 | 24088084.24 | 21886231.55 | 21437615.5 | 19432673 | 0 |
| chr16 | 35792809.08 | 33686623.83 | 29769221 | 28112868 | 0 |
| chr17 | 29525628.56 | 28597492.55 | 27587571 | 26161402 | 0 |
| chr18 | 27491739.43 | 26153894.13 | 24114644 | 23136488.5 | 4.89E-45 |
| chr19 | 21802572.86 | 21449739.36 | 18801773 | 18603477 | 6.09E-106 |
| chr20 | 22331258.79 | 20913415.13 | 19274383 | 17891777.5 | 6.85E-235 |
| chr21 | 8359215.437 | 8481695.516 | 7035295 | 7308014 | 0.04319115366 |
| chr22 | 11141534.4 | 10439516.53 | 9755922 | 9281819 | 3.61E-171 |
| chrX | 52772051.89 | 27195461.17 | 46352309 | 15134665 | 0 |
| all | 57065414.57 | 54455910.19 | 41737711 | 39617204.5 | 0 |

**Supplemental Table** 2. Number and percentage of DM CG sites by chromosome.

|  | CG.Sites | DM | DM% | DM-  hypermethylated | DM-  hypomethylated | %DM  hyper | %DM  hypo |
| --- | --- | --- | --- | --- | --- | --- | --- |
| chr1 | 26352 | 2601 | 9.87% | 1536 | 1065 | 59.05% | 40.95% |
| chr2 | 19322 | 1783 | 9.23% | 1105 | 678 | 61.97% | 38.03% |
| chr3 | 13721 | 1261 | 9.19% | 794 | 467 | 62.97% | 37.03% |
| chr4 | 11023 | 878 | 7.97% | 593 | 285 | 67.54% | 32.46% |
| chr5 | 13912 | 1469 | 10.56% | 958 | 511 | 65.21% | 34.79% |
| chr6 | 20381 | 1513 | 7.42% | 997 | 516 | 65.90% | 34.10% |
| chr7 | 16765 | 1711 | 10.21% | 962 | 749 | 56.22% | 43.78% |
| chr8 | 11286 | 1326 | 11.75% | 634 | 692 | 47.81% | 52.19% |
| chr9 | 5852 | 354 | 6.05% | 247 | 107 | 69.77% | 30.23% |
| chr10 | 13864 | 1302 | 9.39% | 724 | 578 | 55.61% | 44.39% |
| chr11 | 16368 | 1380 | 8.43% | 777 | 603 | 56.30% | 43.70% |
| chr12 | 13592 | 1306 | 9.61% | 725 | 581 | 55.51% | 44.49% |
| chr13 | 6894 | 638 | 9.25% | 387 | 251 | 60.66% | 39.34% |
| chr14 | 8656 | 767 | 8.86% | 487 | 280 | 63.49% | 36.51% |
| chr15 | 8560 | 661 | 7.72% | 393 | 268 | 59.46% | 40.54% |
| chr16 | 12186 | 782 | 6.42% | 459 | 323 | 58.70% | 41.30% |
| chr17 | 15791 | 1097 | 6.95% | 797 | 300 | 72.65% | 27.35% |
| chr18 | 3347 | 340 | 10.16% | 214 | 126 | 62.94% | 37.06% |
| chr19 | 14046 | 983 | 7.00% | 782 | 201 | 79.55% | 20.45% |
| chr20 | 5872 | 584 | 9.95% | 366 | 218 | 62.67% | 37.33% |
| chr21 | 2171 | 165 | 7.60% | 112 | 53 | 67.88% | 32.12% |
| chr22 | 4639 | 274 | 5.91% | 211 | 63 | 77.01% | 22.99% |
| chrX | 8390 | 186 | 2.22% | 90 | 96 | 48.39% | 51.61% |
| All | 272990 | 23361 | 8.56% | 14350 | 9011 | 61.43% | 38.57% |

The first column is the total number of CG sites, and the second and third columns are the number and percentage of differentially methylated sites. The other columns are the numbers and percentages of hypermethylated and hypomethylated sites.

**Supplemental Table** 3. DM CG sites that are highly correlated with a certain number of other sites.

| Interval | 0 | 1-99 | 100-499 | 500-999 | 1000-  4999 | 5000-  9999 | 10k+ | Total |
| --- | --- | --- | --- | --- | --- | --- | --- | --- |
| Tumor (All) | 94984  (34.7939%) | 120871  (44.2767%) | 20073  (7.3530%) | 4852  (1.7774%) | 4872  (1.7847%) | 1913  (0.7008%) | 25425  (9.3135%) | 272990 |
| Normal (All) | 71100  (26.0449%) | 68018  (24.9159%) | 26098  (9.5601%) | 11951  (4.3778%) | 30461  (11.1583%) | 13698  (5.0178%) | 51664  (18.9252%) | 272990 |
| Tumor (0.2) | 6488  (27.7728%) | 14545  (62.2619%) | 1846  (7.9021%) | 431  (1.8450%) | 50  (0.2140%) | 0  (0%) | 1  (0.0043%) | 23361 |
| Normal (0.2) | 5902  (25.2643%) | 8764  (37.5155%) | 4041  (17.2981%) | 1191  (5.0982%) | 1875  (8.0262%) | 760  (3.2533%) | 828  (3.5444%) | 23361 |
| Tumor (0.3) | 1116  (25.3809%) | 2793  (63.5206%) | 383  (8.7104%) | 96  (2.1833%) | 9  (0.2047%) | 0  (0%) | 0  (0%) | 4397 |
| Normal (0.3) | 861  (19.5815%) | 1669  (37.9577%) | 1153  (26.2224%) | 309  (7.0275%) | 295  (6.7091%) | 76  (1.7285%) | 34  (0.7733%) | 4397 |
| Tumor (0.4) | 118  (28.1623%) | 277  (66.1098%) | 21  (5.0119%) | 3  (0.7160%) | 0  (0%) | 0  (0%) | 0  (0%) | 419 |
| Normal (0.4) | 38  (9.0692%) | 161  (38.4248%) | 151  (36.0382%) | 47  (11.2172%) | 22  (5.2506%) | 0  (0%) | 0  (0%) | 419 |

The columns represent the number of high correlations a CG site has in the tumor or normal data, the row name denotes whether the row contains data for the tumor or normal dataset, and the rows represent the degree of the cutoff for differential methylation. The first two rows have no differential methylation cutoff (all 272990 CG sites), with each pair of rows having successively higher estimates for the difference in methylation (p < 0.05, mean difference > 0.2, 0.3, or 0.4).

**Supplemental Table 4**. Summary of the number of CG sites left after testing various filtering criteria.

|  | total.raw | Stdev  ≥0.025 | Stdev  ≥0.05 | Q3-Q1  ≥0.05 | Q3-Q1  ≥0.1 | filt.outliers.coef2 | filt.outliers.coef3 | ≤11NAs | sd0.05.NAfilt.  outliersfiltcoef2 | sd0.05.NAfilt.  outliersfiltcoef3 |
| --- | --- | --- | --- | --- | --- | --- | --- | --- | --- | --- |
| allTCGA.tumor | 476947 | 307538 | 253065 | 222111 | 168908 | 375848 | 396568 | 391229 | 200174 | 211434 |
| allTCGA.normal | 476947 | 262857 | 155811 | 173282 | 72675 | 372935 | 408438 | 391117 | 122284 | 140727 |
|  |  |  |  |  |  |  |  |  |  |  |
|  | total.raw | Stdev  ≥0.025 | Stdev  ≥0.05 | Q3-Q1  ≥0.05 | Q3-Q1  ≥0.1 | filt.outliers.coef2 | filt.outliers.coef3 | ≤11NAs | sd0.05.NAfilt.  outliersfiltcoef2 | sd0.05.NAfilt.  outliersfiltcoef3 |
| allTCGA.tumor | 100.00% | *64.48%* | 53.06% | 46.57% | 35.41% | **78.80%** | **83.15%** | **82.03%** | 41.97% | 44.33% |
| allTCGA.normal | 100.00% | *55.11%* | 32.67% | 36.33% | 15.24% | **78.19%** | **85.64%** | **82.00%** | 25.64% | 29.51% |

Upper table gives the number of CG sites, while the lower table gives the proportion of CG sites. “stddev” refers to the standard deviation, and “Q3-Q1” refers to the Interquartile Range. “Filt.outliers” refers to filtering out those sites that have 1 or 2 outliers, with either coef=2 or coef=3. Coef refers to how many IQRs away a point must be from Q1 or Q3 to be considered an outlier. Hence, coef=2 means an outlier must be 2 IQRs away, and coef=3 means an outlier must be 3 IQRs away.
